# Moderate organic–inorganic fertilization optimizes soybean productivity by reshaping rhizosphere microbiome–metabolite networks

**DOI:** 10.3389/fpls.2026.1823609

**Published:** 2026-06-01

**Authors:** Jing Zhang, Qiang Liu, Jiali Chen, Yongmei Zhou, Bianhong Zhang, Zhaonian Yuan, Peiwu Li, Ziqin Pang

**Affiliations:** 1 Xianghu Laboratory, Hangzhou, China; 2 College of Natural Resources and Environment, Northwest Agriculture and Forestry University, Yangling, Shaanxi, China; 3 Fujian Key Laboratory of Agroecological Processing and Safety Monitoring, Fujian Agriculture and Forestry University, College of Agriculture, Fuzhou, China; 4 National Engineering Research Center for Sugarcane, Fujian Agriculture and Forestry University, Fuzhou, China

**Keywords:** metabolome reprogramming, organic-inorganic fertilization, rhizosphere microbiome, soil health, soybean, sustainable fertilization, symbiotic interactions, yield optimization

## Abstract

Soybean, a key oilseed and fodder crop, is pivotal for national food security in China. And sustainable soybean production requires fertilization strategies that enhance yield while restoring rhizosphere ecological function. Balancing chemical and organic fertilization is crucial for sustainable soybean production, yet the underlying rhizosphere mechanisms driving crop performance remain underexplored. We conducted a nutrient-equalized gradient substitution experiment comparing chemical fertilizer (CF) with 30%, 70%, and 100% organic fertilizer replacement (OF30, OF70, OF100), investigating the effects on soybean growth, rhizosphere soil properties, bacterial and fungal microbiomes, and metabolomes, while maintaining equivalent nutrient inputs. Moderate organic-inorganic fertilization (30/70% organic substitution, designated as OF30 and OF70) significantly enhanced plant height, root length, biomass, nodulation, nitrogenase activity, photosynthetic capacity, and yield compared to full chemical fertilization (CF, 0% organic) and full organic fertilization (OF100%, 100% organic), the application of 30% organic and 70% inorganic fertilization in combination identified as the optimal strategy. These gains suggest that rhizosphere soil exhibited improved pH, organic carbon, and nutrient availability (K and P), alongside balanced nitrogen. Bacterial communities showed conserved core structure but increased α-diversity and turnover toward metabolically versatile genera (e.g., *Flavobacterium*, *Geobacter*, *Luteibacte*r) under organic-inorganic fertilization. Fungal assemblages preserved a stable core while enriching saprotrophic and beneficial guilds (e.g., *Serendipita*, *Chaetomium*, *Arthrobotrys*). Metabolomics revealed conserved profiles with targeted enrichment of carbon-related classes (e.g., glycerophospholipids, flavonoids like delphinidin), supporting microbial activity and plant-microbe signaling. Integrated analyzes indicated that moderate organic substitution (30/70%) reshapes the rhizosphere toward balanced nutrient cycling, enhanced microbiome diversity, and functional metabolite pools, fostering symbiotic interactions and improving nutrient availability. These findings highlight moderate organic-inorganic blending as an optimal strategy for improving soybean productivity and soil health, with implications for sustainable cropping systems.

## 1 Introduction

Soybean (*Glycine max*) is a cornerstone of global food security, plant protein supply, and sustainable agroecosystems, owing to its high nutritional value and its capacity to improve soil fertility through biological nitrogen inputs (Zhang et al., 2025). As soybean cultivation expands to meet rising global demand, maintaining yield stability while preserving soil health has become an increasingly urgent challenge (Wulanningtyas et al., 2021; Song et al., 2024). Rhizosphere engineering, through tailored fertilization regimes and targeted microbial inoculants, offers a promising strategy to improve nutrient use efficiency by reshaping root-microbe communication and rhizosphere function (Zhang et al., 2010; Saad et al., 2020; Ji et al., 2023; Griffin et al., 2024; Lestari et al., 2024).

Organic fertilizers derived from manure, compost, or humic substances are widely promoted as sustainable alternatives to mineral fertilizers, given their capacity to increase soil organic carbon, enhance nutrient retention, and stimulate microbial activity (Wu et al., 2019; Yan et al., 2021). However, full replacement of mineral fertilizers with organic inputs does not consistently improve crop yield, likely due to slower nutrient mineralization, mismatched nutrient stoichiometry, or transient nutrient limitations (Reganold and Wachter, 2016; Wang et al., 2024d). Increasing evidence suggests that partial organic-inorganic fertilizer substitution often outperforms either input alone, indicating that crop productivity depends not merely on nutrient quantity, but on coordinated carbon-nutrient-microbiome interactions (Samson et al., 2020; Wang et al., 2026).

Recent advances in rhizosphere ecology have reframed fertilization as a systems-level intervention that reshapes soil biogeochemistry, microbial community assembly, and metabolite-mediated signaling networks (Tong et al., 2025; Xing et al., 2025). Soil nutrient stoichiometry and carbon availability are now recognized as key ecological filters governing microbial recruitment, functional redundancy, and ecosystem stability (Fierer, 2017; Delgado-Baquerizo et al., 2018; Yan et al., 2025). Organic amendments, in particular, can promote metabolically versatile bacterial taxa, expand microbial diversity, and enhance nutrient-mobilization capacity (Shu et al., 2022; Sun et al., 2025; Yang et al., 2025). In parallel, rhizosphere metabolomics has revealed that plants and microbes co-construct chemical landscapes enriched in organic acids, sugars, flavonoids, and secondary metabolites that regulate microbial selection, redox balance, hormone signaling, and nutrient acquisition (Sasse et al., 2018; Zhalnina et al., 2018; Ling et al., 2022).

This systems perspective has been further advanced by the demonstration that synthetic microbial communities can couple pathogen suppression with symbiotic nitrogen fixation, achieving aflatoxin reduction and super-nodulation without yield penalty through coordinated carbon allocation and microbial recruitment (Zhang et al., 2026b).

Despite these conceptual advances, a major mechanistic gap remains: How does organic-inorganic fertilization coordinately restructure soil chemistry, rhizosphere metabolite composition, and cross-kingdom microbiome assembly, and how do these coupled processes translate into improved crop growth and yield?

Most previous studies have examined soil nutrients, microbiomes, or metabolites independently, limiting the ability to resolve causal linkages across trophic and biochemical layers (Hartmann et al., 2015; Bhattacharjya et al., 2024). Moreover, few studies have tested nutrient-equalized organic substitution gradients, making it difficult to disentangle fertilization strategy effects from total nutrient input (Hartman et al., 2017; Xu et al., 2024; Nie et al., 2025). As a result, the non-linear productivity response to partial organic substitution remains mechanistically unexplained, and the theoretical basis for optimizing organic-inorganic fertilization is still underdeveloped.

Legume cropping systems offer a particularly powerful model for addressing this question. Soybean productivity is tightly coupled to nitrogen availability, root metabolic status, and rhizosphere microbial function (Salvagiotti et al., 2008; Ferguson et al., 2019; Lian et al., 2023; Yan and Bisseling, 2024). Excess mineral nitrogen is known to suppress nodulation and disrupt nitrogen-related signaling pathways (Streeter, 1985; Wang et al., 2020; Lin et al., 2021), whereas organic amendments may stabilize nutrient supply and enhance microbial-mediated nutrient turnover (Shu et al., 2022; Bai et al., 2026). Yet, the integrated soil-metabolite-microbiome-root function-growth-yield cascade has rarely been resolved within a single experimental design, limiting progress toward predictive, microbiome-informed fertilization models.

Here, we conducted a nutrient-equalized gradient substitution experiment, comparing chemical fertilization (CF, 0% organic) with low-to-moderate (OF30, 30% organic), moderate-to-high (OF70, 70% organic), and full (OF100, 100% organic) organic fertilizer replacement derived from chicken manure and humic acid. By integrating plant phenotyping, soil chemistry, bacterial and fungal community profiling, and rhizosphere metabolomics, we aimed to: identify the agronomic optimum of organic-inorganic fertilization; characterize microbial and metabolite signatures associated with enhanced growth and yield; and construct a mechanistic framework describing how organic inputs reorganize rhizosphere biogeochemical and biological networks.

We demonstrate that moderate organic substitution (30/70%) maximizes soybean growth and yield, coinciding with soil carbon enrichment, nutrient rebalancing, metabolite reprogramming, and coordinated bacterial-fungal restructuring. These findings support a causal soil, metabolite, microbiome, and growth cascade, advancing a conceptual model in which fertilization functions as an ecological lever that rhizosphere carbon distribution and microbial functional capacity. By integrating multi-omics and plant performance metrics, this study provides a mechanistic foundation for microbiome-informed fertilization strategies, with implications for sustainable soybean production and broader agroecosystem management.

## 2 Materials and methods

### 2.1 Plant materials and growth conditions

**Figure 1:**
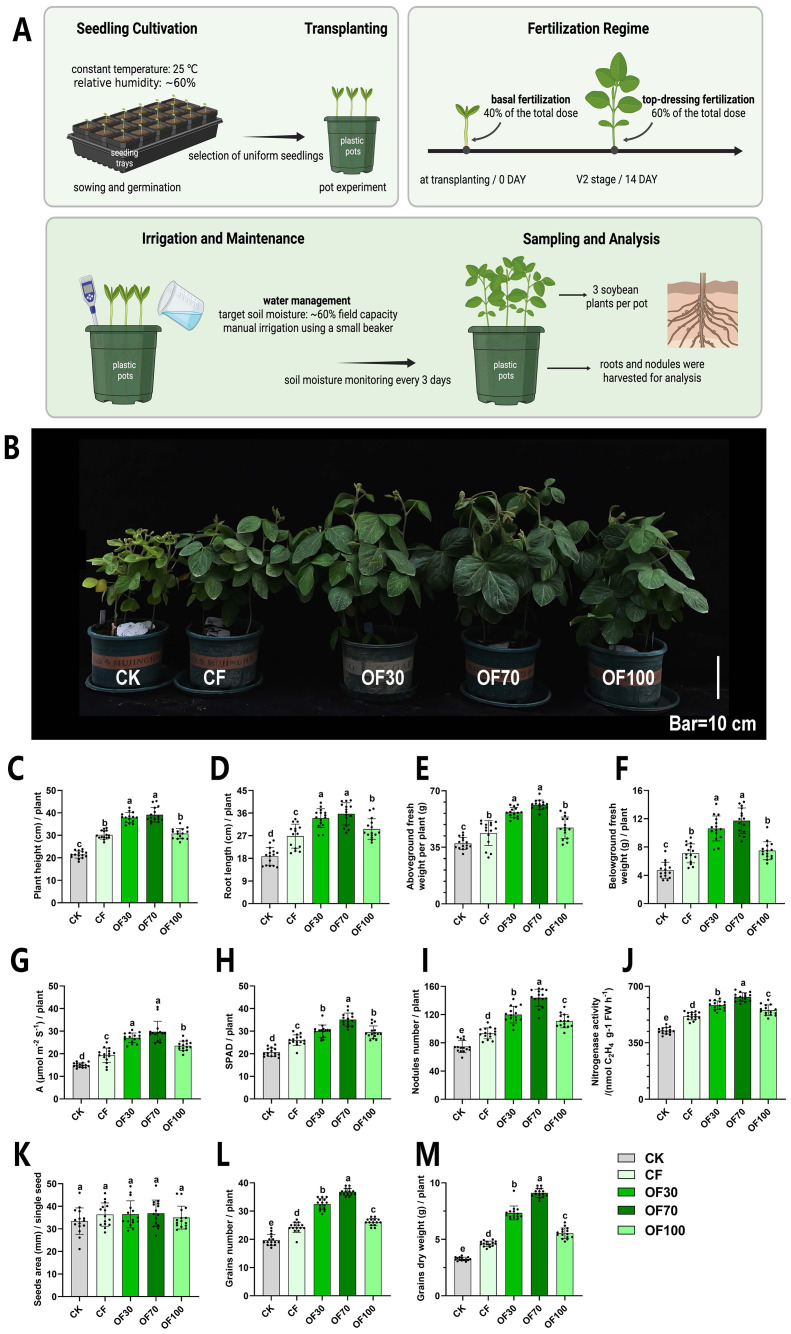
Organic-inorganic fertilizer substitution enhances soybean growth, nodulation, photosynthetic performance, and yield formation. **(A)** Schematic illustration of the soybean pot cultivation system, including seedling culture, transplanting, split fertilization, moisture management, and sampling procedures. **(B)** Phenotypic performance of soybean plants at the anthesis stage under different fertilization treatments (scale bar = 10 cm); **(C)** Plant height per plant; **(D)** Root length per plant; **(E)** Aboveground fresh weight per plant; **(F)** Belowground fresh weight per plant; **(G)** Net photosynthetic rate **(A)**; **(H)** Leaf chlorophyll content (SPAD value); **(I)** Nodule number per plant; **(J)** Nitrogenase activity per gram of fresh nodule weight; **(K)** Surface area of a single dry seed at harvest; **(L)** Total seeds number per plant at harvest; **(M)** Total dry seeds weight per plant at harvest. Different lowercase letters indicate significant differences among treatments (*P* < 0.05). CK, no fertilizer; CF, chemical fertilizer only (N–P_2_O_5_–K_2_O); OF30 and OF70, 30% and 70% substitution of inorganic fertilizer with organic fertilizer, respectively; OF100, 100% organic fertilizer derived from chicken manure and humic acid.

The experiment was conducted in the greenhouse at Fujian Agriculture and Forestry University, Fuzhou, Fujian Province, China (119° 14′ 36″E, 26° 5′ 18″N). The experimental site is located in a subtropical humid monsoon climate zone, with a mean annual temperature of approximately 21.1 °C. The commonly cultivated local soybean (*Glycine max* L.) variety “MinDou No.5” was used as the test material. And “MinDou No.5” is a high-yielding vegetable soybean cultivar with wide adaptability in Fujian Province, showing strong nodulation ability and moderate growth period. Its average fresh pod yield is 9960.6 kg·hm^-2^ in regional trials (Hu et al., 2014). A pot cultivation system was employed in this study. Soybean seeds were first sown in seedling trays with nutrient soil. The greenhouse environment was maintained at a constant temperature of 25 °C and a relative humidity of approximately 60%. After one week, uniform seedlings were selected and transplanted into plastic cultivation buckets (specifications: inner diameter 25 cm, height 12 cm). The five treatments were applied in a split-application strategy. Specifically, 40% of the total fertilizer dose was applied as basal fertilizer, and the remaining 60% was supplied at the soybean V2 (second trifoliolate, 14 days after transplanting) stage. Subsequent pot management practices, including regular watering and routine plant maintenance, were carried out under greenhouse conditions according to the experimental design. Soil moisture was monitored every 3 days and maintained at 60%-70% of field capacity by artificial watering (small beaker irrigation) based on leaf weight variation (**Figure 1A**). The experimental soil was classified as sandy loam with suitable fertility for soybean growth, and its physicochemical properties prior to the experiment were as follows: pH 6.53, available nitrogen (AN) 43.75 mg·kg^-1^, soil organic carbon (SOC): 14.61 g·kg^-1^, available phosphorus (AP) 17.99 mg·kg^-1^, and available potassium (AK) 82.63 mg·kg^-1^.

### 2.2 Experimental design and sampling strategy

Five fertilization treatments were established to evaluate the effects of different organic-inorganic fertilizer substitution ratios: CK (no fertilizer control), CF (chemical fertilizer only, 0% organic substitution), OF30 (low-to-moderate organic substitution, 30% organic + 70% chemical fertilizer), OF70 (moderate-to-high organic substitution, 70% organic + 30% chemical fertilizer), and OF100 (full organic substitution, 100% organic fertilizer + 0% chemical fertilizer). The OF30 and OF70 treatments represent moderate organic-inorganic fertilization, where organic and inorganic nutrients are combined in balanced proportions, contrasting with the extreme inputs of pure chemical (CF) or pure organic (OF100) fertilization. This classification follows established frameworks in sustainable fertilization research, where moderate organic substitution typically ranges from 25% to 75% depending on crop type and soil conditions (Zhao et al., 2020; Wu et al., 2024a; Nie et al., 2025). Each treatment consisted of three biological replicates. Detailed compositions and application rates of all fertilizers are provided in **Table 1**. The total nutrient input was equalized across all fertilization treatments at 450 kg·hm^-2^ (N + P_2_O_5_ + K_2_O), based on the standard fertilization rate for vegetable soybean cultivation in Fujian Province (Hu et al., 2014). This equalized design was achieved by adjusting the application rates of organic and chemical fertilizers according to their respective nutrient contents. The specific application rates for each treatment are: CF (chemical fertilizer only): 450 kg·hm^-1^; OF30: 1200 kg·hm^-2^ organic + 315 kg·hm^-2^ chemical; OF70: 2800 kg·hm^-2^ organic + 135 kg·hm^-2^ chemical; OF100: 4000 kg·hm^-2^ organic fertilizer. The organic fertilizer was purchased from a commercial supplier (product standard: NY 525-2012, number of invention patent:ZL201410263153.5). And the organic fertilizer was chicken manure-based. Regarding the nutrient equalization, the substitution percentages (30%, 70%, 100%) refer to the proportion of total nutrient supply contributed by each fertilizer type, not the simple weight ratio. This proportional substitution design follows established methodologies in organic-inorganic fertilization studies (Zhao et al., 2020; Wu et al., 2024a). And the applied organic fertilizer was dry granular organic fertilizer with a moisture content <35% (as specified in **Table 1**). The chemical fertilizer was also in granular form. Both fertilizers were applied as solid amendments, not liquid formulations. Each treatment consisted of three biological replicates. Detailed application rates per pot and per hectare are provided in **Table 1**. Rhizosphere soil samples (soil tightly adhering to the root surface) were collected at the soybean harvest stage (90 days after sowing), using the shake-off method as described (Liu et al., 2025). The collected soil from each replicate was homogenized and subsequently divided into two subsamples. One portion was air-dried at room temperature for the determination of soil physicochemical properties, while the other portion was immediately stored at −80 °C for soil DNA extraction and metabolite profiling, following the procedures outlined (Wang et al., 2024a).

**Table 1:** A gradient substitution design of organic fertilizer for chemical fertilizer.

Treatments	(N+P_2_O_5_+K_2_O≥5.0%, organic matter ≥ 45%) (N-P-K 1.7-2.6-1.4, water content<35%)	(N-P-K 10-15-12)
	Organic Fertilizer (g/pot), (kg/ha)	Chemical fertilizer (g/pot), (kg/ha)
CK	0	0
CF	0 (0%)	22.5 (100%), 450 (100%)
OF30	60 (30%), 1200 (30%)	15.75 (70%), 315 (70%)
OF70	140 (70%), 2800 (70%)	6.75 (30%), 135 (30%)
OF100	200 (100%), 4000 (100%)	0 (0%)

The chemical fertilizer was compound fertilizer with N-P_2_O_5_-K_2_O = 10-15-12 (10% N, 15% P_2_O_5_, 12% K_2_O per kg). The organic fertilizer was commercially produced dry granular fertilizer derived from fermented chicken manure, supplemented with humic acid, with total nutrient content N+P_2_O_5_+K_2_O ≥ 5.0% (specifically N-P_2_O_5_-K_2_O = 1.7-2.6-1.4, i.e., 1.7% N, 2.6% P_2_O_5_, 1.4% K_2_O per kg) and organic matter ≥ 45%. The organic fertilizer was purchased from a commercial supplier (product standard: NY 525-2012,number of invention patent:ZL201410263153.5). The organic fertilizer was chicken manure-based. Regarding the nutrient equalization: the substitution percentages (30%, 70%, 100%) refer to the proportion of total nutrient supply contributed by each fertilizer type, not the simple weight ratio. For example, OF30 indicates that organic fertilizer contributes 30% of the total nutrient input, with chemical fertilizer contributing the remaining 70%. This proportional substitution design follows established methodologies in organic-inorganic fertilization studies (Zhao et al., 2020; Wu et al., 2024a). And the applied organic fertilizer was dry granular organic fertilizer with a moisture content <35%. The chemical fertilizer was also in granular form. Both fertilizers were applied as solid amendments, not liquid formulations.

All data are presented as mean ± standard error. Lowercase letters indicate the significance of the difference based on the Games-Howell test (*P* < 0.05).

### 2.3 Assessment of soybean growth parameters

Plant physiological traits and root nodule phenotypes were measured to evaluate treatment effects on soybean growth and symbiotic performance. Leaf chlorophyll content was quantified using a portable SPAD-502 Plus chlorophyll meter (Konica Minolta, Japan), which estimates relative chlorophyll concentration based on dual-wavelength optical absorbance. Net photosynthetic rate was measured using a portable photosynthesis system (LI-680, LI-COR, USA). Both measurements were conducted on the third fully expanded leaf from the top of each plant between 9:00 and 11:00 a.m., with three biological replicates as described (Xun et al., 2024). Root and nodule phenotypes were assessed at the indicated time points using a microscope equipped with a digital camera (GXY-A plus, Top Cloud-agri, China).

### 2.4 Measure the activity of nitrogenase

An acetylene reduction activity (ARA) assay was used to measure the nitrogenase activity of root nodules as described previously described (Montes-Luz et al., 2023), with modifications implemented in this work. Soybean fresh nodules immediately placed into sealed 60 mL glass vial. To initiate the reaction, 3 mL of air was withdraw from each vial and replaced with 3 ml of acetylene. The vials were then inverted in a water bath to ensure airtight conditions and incubated at 28 °C for 2 h. Reactions were terminated by chilling the vials on ice. A 3 mL gas sample from each vial was analyzed for ethylene production using a gas chromatograph (Nexis GC-2030, Daojing, Japan) equipped with a PLOT/Q capillary column (30 m × 0.32 mm × 0.25 μm) and a flame-ionization detector operated at 250 °C. Nitrogen was used as the carrier gas (2.0 mL/min), and hydrogen and air flow rates were set at 32 mL/min and 200 mL/min, respectively. Nitrogenase activity was normalized to nodule fresh weight and/or per plant. For each assay, nodules from at least 15 individual soybean plants were analyzed. Each pot was treated as one biological replicate, and nodules from 3 plants within the same pot were averaged for analysis. Significant differences among treatments were analyzed in R using Welch’s ANOVA followed by Games-Howell *post hoc* test, with *P* < 0.05 considered statistically significant.

### 2.5 Analysis the rhizosphere soil physicochemical characteristics

Air-dried soil samples were used to determine baseline physicochemical properties following standardized protocols. Soil pH was measured in a 1:2.5 (w/v) soil–water suspension using a calibrated pH meter (PHS-3C, INESA Scientific Instrument Co., Ltd., Shanghai, China). Available nitrogen (AN) was quantified using the alkaline hydrolyzable diffusion method. Available potassium (AK) was extracted with 1 M ammonium acetate and determined by flame photometry, while available phosphorus (AP) was extracted using sodium bicarbonate and measured by the molybdenum blue colorimetric method. All analyzes were conducted in accordance with the procedures described (Chen et al., 2020).

### 2.6 DNA extraction and high-throughput sequencing of bacterial communities

0.5 g of rhizosphere soil genomic DNA was extracted using the PowerSoil DNA Isolation Kit (MoBio Laboratories Inc., Carlsbad, CA, USA), which is optimized for inhibitor-rich environmental samples. DNA concentration and purity were quantified using a NanoDrop 2000 spectrophotometer (Thermo Scientific, Waltham, MA, USA). The V3-V4 hypervariable region of the bacterial 16S rRNA gene was amplified using the primer pair 338F (5′-ACTCCTACGGGAGGCAGCAG-3′) and 806R (5′-GGACTACHVGGGTWTCTAAT-3′), validated for profiling diverse soil bacterial communities (Fadrosh et al., 2014).And the PCR program is pre-denaturation at 95 °C for 3 min; 35 cycles of 95 °C for 30 s, 55 °C for 30 s, 72 °C for 45 s; extension at 72 °C for 10 min. Sample-specific barcodes and Illumina sequencing adapters were incorporated into primers prior to amplification. PCR reactions were performed in triplicate to minimize amplification bias, pooled, and purified using a magnetic bead-based cleanup system prior to library construction. Sequencing libraries were generated according to Illumina standard protocols and sequenced on the Illumina HiSeq platform (Illumina Inc., San Diego, CA, USA) using paired-end chemistry with an insert-size of approximately 469 bp. The raw sequencing data are available in the National Center for Biotechnology Information Sequence Read Archive under accession PRJNA1453983. Raw sequencing data were demultiplexed and converted to FASTQ format through Illumina base-calling software (Liu et al., 2023b). Quality control of raw reads was conducted using Trimmomatic (v0.33) to remove low-quality bases and adapter contamination, followed by primer trimming using Cutadapt (v1.9.1). Paired-end reads were merged based on sequence overlap, and chimeric sequences were identified and removed using USEARCH (v10.0) with the UCHIME algorithm to reduce artifacts derived from PCR amplification. Microbial richness (ACE) and diversity indices (Shannon) were calculated using the bioinformatics software QIIME. High-quality sequences were clustered into operational taxonomic units (OTUs) at a 97% sequence similarity threshold using USEARCH (v10.0). To reduce noise from rare sequencing artifacts, OTUs representing <0.005% of total reads were filtered out prior to downstream analyzes (Bokulich et al., 2013). Representative OTU sequences were taxonomically classified against the SILVA rRNA reference database using a naïve Bayesian classifier, with confidence thresholds applied to ensure robust taxonomic assignments (Edgar, 2013).

### 2.7 Metabolite extraction and LC-MS/MS analysis

Samples were thawed on ice (4 °C). And 100 mg of rhizosphere soil was used for metabolite extraction. A 100 μL aliquot of each sample was mixed with 300 μL methanol and 20 μL internal standard (L-chlorophenylalanine, 0.1 mg·mL^-1^) in an EP tube, vortexed for 30 s, sonicated on ice for 10 min, and incubated at -20 °C for 1 h to precipitate proteins. After centrifugation at 13,000 rpm for 15 min (4 °C), 200 μL supernatant was transferred to LC-MS vials. QC samples were prepared by pooling 20 μL aliquots from each extract. Metabolomic profiling was performed via UHPLC-QTOF-MS using an Agilent 1290 UHPLC system coupled with an AB Sciex TripleTOF 5600+ mass spectrometer. Chromatographic separation was achieved on a Waters UPLC BEH Amide column (2.1 × 100 mm, 1.7 μm) with mobile phases A (25 mM NH_4_OAc + 25 mM NH_4_OH in water, pH 9.75) and B (acetonitrile), using the following gradient: 0–7 min, 95%-65% B; 7–9 min, 65%-40% B; 9.1–12 min, 95% B (flow rate: 0.5 mL min^-1^, injection volume: 3 μL). Mass spectrometric data were acquired in information-dependent acquisition (IDA) mode (Tian et al., 2025): full-scan MS spectra triggered MS/MS of the top 12 precursor ions (intensity threshold: 100 counts) with collision energy 30 V and product-ion accumulation time 50 ms. ESI parameters: gas 1/gas 2 = 60 psi, curtain gas = 35 psi, source temperature = 650 °C, ISVF = +5000 V/−4000 V (positive/negative modes). Raw data were processed for peak detection, retention-time alignment, and intensity extraction. Peak annotation was performed using CAMERA, and metabolites were identified via an in-house MS² spectral database (Chong and Xia, 2018).

### 2.8 Multivariate co-occurrence network analysis

Amplicon sequencing data (16S rRNA and ITS) and metabolite datasets were first processed to extract relative abundance matrices at selected taxonomic and metabolite class levels (Fallah et al., 2026). Rhizosphere soil total DNA was extracted from approximately 0.5 g of fresh soil using the PowerSoil DNA Isolation Kit (MoBio Laboratories Inc., Carlsbad, CA, USA), DNA concentration and purity were quantified using a NanoDrop 2000 spectrophotometer (Thermo Scientific, Waltham, MA, USA). The fungal ITS1 region was amplified using the primer pair ITS1F (5′-CTTGGTCATTTAGAGGAAGTAA-3′) and ITS2 (5′-GCTGCGTTCTTCATCGATGC-3′). PCR amplification was initial denaturation at 95 °C for 3 min, followed by 30 cycles of 95 °C for 30 s, 55 °C for 30 s, and 72 °C for 45 s, with a final extension at 72 °C for 10 min. PCR products were purified, quantified, and pooled in equimolar concentrations, and sequencing was conducted on the Illumina HiSeq platform (Illumina Inc., San Diego, CA, USA) using paired-end chemistry with an insert-size of approximately 230 bp (Celestina et al., 2019). Multi-omics matrices were then merged using the merge16S_ITS_meta function to generate a unified dataset. Environmental variables and agronomic traits were screened with env_level, retaining the top 30 factors. Spearman correlations among microbial taxa, metabolite features, and environmental/agronomic variables were calculated using corBiostripe2, and significant links were defined as *P* < 0.05 and |r| > 0.6. The filtered matrix was used to construct a co-occurrence network, with node positions determined via the PolygonRrClusterG2 layout and visualized using the Multi_analyzeplot. This analysis identified key cross-kingdom associations linking microbiome structure, metabolite profiles, soil conditions, and soybean performance across fertilization regimes.

### 2.9 Integrated Pearson-Mantel association analysis of microbial communities, environmental factors, and plant phenotypes

Associations between microbial community composition and environmental variables, crop phenotypic traits, and metabolite classes were evaluated using Mantel tests implemented in the linkET package (GitHub repository: https://github.com/Hy4m/linkET) in R. Pearson correlation coefficients among environmental, phenotypic, and metabolite variables were calculated and visualized as correlation heatmaps using ggplot2. Mantel test results were subsequently integrated into the heatmap framework as overlaid network links to illustrate cross-domain associations. Color schemes were optimized using the cols4all package to ensure visual clarity and interpretability. Correlation coefficients and Mantel statistics (r and P values) were exported as summary tables (**Supplementary Table 4**) for downstream analysis and reporting.

### 2.10 Analysis methods

Bacterial functional guilds were inferred from 16S rRNA gene-based taxonomic data using FAPROTAX (Pang et al., 2021). Relative abundances of functional guilds was calculated for rhizosphere soil samples (n = 3). Differences between treatments were assessed using Welch’s t-test with false discovery rate correction, and results were visualized as extended error bar plots showing means and 95% confidence intervals. Significant one-way ANOVA and Tukey’s honestly significant difference (HSD) *post hoc* tests were performed in R to assess intergroup differences in soybean agronomic traits, nitrogenase activity, and rhizosphere soil physicochemical properties. Microbial richness (ACE) and diversity indices (Shannon) were calculated using the bioinformatics software QIIME. Principal Coordinates Analysis (PCoA) based on Bray-Curtis dissimilarity was performed using the vegan package in R. To visualize the taxonomic composition at the bacterial phylum level and HMDB-based metabolite classification, the functions of the reshape2, ggplot2, and vegan packages in R were integrated to generate overlay plots (Zhang et al., 2019). Differential abundance analysis among treatment groups was carried out using the DESeq2 package in R, followed by detailed visualizations including ternary diagrams created with ggpubr, ggrepel, ggplot2, grid, and ggraph packages, providing intuitive graphical representations of the statistical differences (Pang et al., 2022).

## 3 Results

### 3.1 Organic-inorganic fertilization enhances soybean growth, nodulation, photosynthetic capacity, and yield

A gradient substitution experiment was established to evaluate the effects of replacing chemical fertilizer with organic fertilizer, ranging from 0% (CF) to 100% substitution (OF100), while maintaining comparable nutrient inputs across treatments (**Table 1**). This design enabled the disentanglement of fertilization strategy effects from total nutrient supply (**Figure 1A**).

Across treatments, progressive substitution of chemical fertilizer with organic fertilizer resulted in substantial improvements in soybean growth performance (**Figure 1B**). Compared with CF, OF30 and OF70 significantly increased plant height and root length (**Figures 1C, D**), indicating enhanced shoot and root development. Biomass accumulation followed a similar trend, with aboveground and belowground fresh weights peaking under OF70 (**Figures 1E, F**), suggesting improved assimilated carbon allocation and plant vigor under OF30 and OF70 substitution. Net photosynthetic rate and chlorophyll content were significantly higher in OF30 and OF70 relative to CF, reflecting improved leaf physiological status and photosynthetic capacity (**Figures 1G, H**). These increases likely contributed to the observed improvements in biomass production and reproductive output.

Organic-inorganic fertilization further exerted strong positive effects on nodulation and symbiotic nitrogen fixation. The number of nodules per plant increased significantly in OF30 and OF70, accompanied by a pronounced rise in nitrogenase activity per gram of fresh nodule tissue (**Figures 1I, J**). And nodule number was significantly positively correlated with nitrogenase activity (r = 0.65, *P* < 0.01), indicating that enhanced nodulation directly supports greater symbiotic nitrogen fixation capacity. Yield-related traits exhibited consistent improvements under organic fertilizer treatments. Except for seed surface area, total seed number per plant, and total dry seed weight were significantly elevated in OF30 and OF70 compared with CF (**Figures 1K–M**). Among all treatments, OF70 consistently achieved the highest yield performance, confirming that moderate organic substitution (30/70%) optimizes soybean productivity, whereas full organic substitution (OF100) did not further increase yield. Actually, OF100 lowered yield relative to OF30 and OF70. We would not ignore this result, potentially important. This may be resulted from the reduced CF input, suggesting a CF-OF balance required for optimal yield.

Collectively, these results indicate that partial substitution of chemical fertilizer with organic fertilizer, particularly at 30/70% replacement enhances soybean vegetative growth, nodulation efficiency, photosynthetic performance, and yield formation, outperforming conventional chemical fertilization while maintaining equivalent nutrient inputs.

### 3.2 Organic fertilizer substitution improves rhizosphere soil fertility and nutrient availability

To determine whether improved soybean performance was accompanied by changes in rhizosphere soil properties, key soil chemical parameters were quantified across fertilization treatments (**Table 2**).

**Table 2:** The application of different fertilizers influenced rhizosphere soil fertility.

Treatment	pH	AN(mg/kg) Available nitrogen	AK(mg/kg) Available potassium	AP(mg/kg) Available phosphorus	SOC(g/kg) Soil organic carbon
CK	6.19±0.12a	37.33±3.09b	62.54±5.13cd	14.02±1.76bc	13.91±1.06c
CF	5.46±0.10b	53.67±9.55a	58.37±2.14d	22.35±0.62a	15.99±0.56bc
OF30	5.75±0.33a	57.63±13.56a	84.99±6.73b	19.96±1.76ab	16.44±0.64b
OF70	5.70±0.04a	49.00±2.02a	114.36±5.12a	17.76±3.25abc	17.70±0.93ab
OF100	5.91±0.12a	48.53±3.76a	79.88±10.57bc	13.50±1.88c	19.21±0.25a

All data are presented as mean ± standard error. Lowercase letters indicate the significance of the difference based on the LSD test (*P* < 0.05).

Organic fertilizer substitution altered rhizosphere soil pH, increased nutrients availability and organic carbon content. CF resulted in the lowest soil pH, whereas organic fertilizer treatments increased pH, with the highest values observed under OF100. Substitution levels at OF30 and OF70 showed moderate but significant pH recovery relative to CF.

Available nitrogen (AN) displayed a non-linear response to organic substitution. CF exhibited the highest AN, while increasing organic fertilizer input reduced AN levels, with OF30 and OF70 maintaining intermediate nitrogen availability and OF100 showing the lowest AN concentration. Organic fertilizer substitution markedly increased soil available potassium (AK) and phosphorus (AP). AK was highest under OF70, significantly exceeding CF and OF30, while AP was also elevated under organic fertilizer treatments, with OF70 showing the greatest increase. Soil organic carbon (SOC) increased progressively with organic fertilizer substitution, with OF100 showing the highest SOC, followed by OF70 and OF30, all exceeding CF.

Overall, organic fertilizer substitution reshaped rhizosphere soil fertility by optimizing nitrogen availability, enhancing potassium and phosphorus pools, and elevating soil organic carbon, with OF30/OF70 exhibiting a more balanced nutrient profile.

### 3.3 Organic fertilizer substitution preserves a stable core rhizosphere microbiome while subtly reshaping peripheral diversity

High-throughput sequencing generated approximately 1.20 million raw paired-end reads across all rhizosphere samples, with ~77,000–80,000 reads per library (**Supplementary Table 1**). After quality filtering and chimera removal, 1.13 million high-quality reads were retained for downstream analyzes. Read length (417–420 bp) and GC content (53.7–56.9%) were consistent across samples, and sequencing quality was high, with Q20 > 98.9%, Q30 > 95.8%, and effective read rates generally exceeding 95% (**Supplementary Table 1**). Rarefaction curves reached clear plateaus for all samples (**Supplementary Figure 1**), indicating sufficient sequencing depth to capture rhizosphere bacterial diversity.

At the soybean harvest stage, rhizosphere bacterial communities across all treatments were dominated by *Proteobacteria* (26.19–29.57%), *Acidobacteria* (15.29–24.70%), *Bacteroidetes* (8.60–15.52%), *Firmicutes* (6.15–16.32%), and *Chloroflexi* (4.63–8.96%), with *Nitrospirae* (1.37–1.97%) consistently present at lower abundance. Along the organic substitution gradient, Proteobacteria showed a modest increase under OF treatments relative to CK and CF, whereas Acidobacteria tended to be higher in OF30 and OF100. In contrast, Bacteroidetes, Firmicutes, and Chloroflexi displayed a slight decline at higher organic input (OF100). Overall, phylum-level composition remained broadly conserved, indicating that organic substitution primarily fine-tunes dominant lineages rather than restructuring the community at deep taxonomic levels (**Supplementary Figure 2**). Venn diagram revealed a large shared core microbiome across all treatments, with 2172 OTUs common to CK, CF, OF30, OF70, and OF100. Total richness was comparable among regimes (2201–2224 OTUs), with OF30 and OF70 showing slightly higher OTU counts than CF and CK, whereas OF100 did not further increase richness. Treatment-specific OTUs were minimal (≤3 per treatment), indicating limited unique taxa (**Supplementary Figure 3**).

Collectively, these data indicate that organic fertilizer substitution primarily modulates low-abundance peripheral taxa while maintaining a highly conserved core microbiome structure, consistent with a buffering effect of moderate organic inputs on rhizosphere bacterial community assembly.

### 3.4 Organic-inorganic fertilization influences directional bacterial community turnover and increases taxonomic richness and evenness

**Figure 2:**
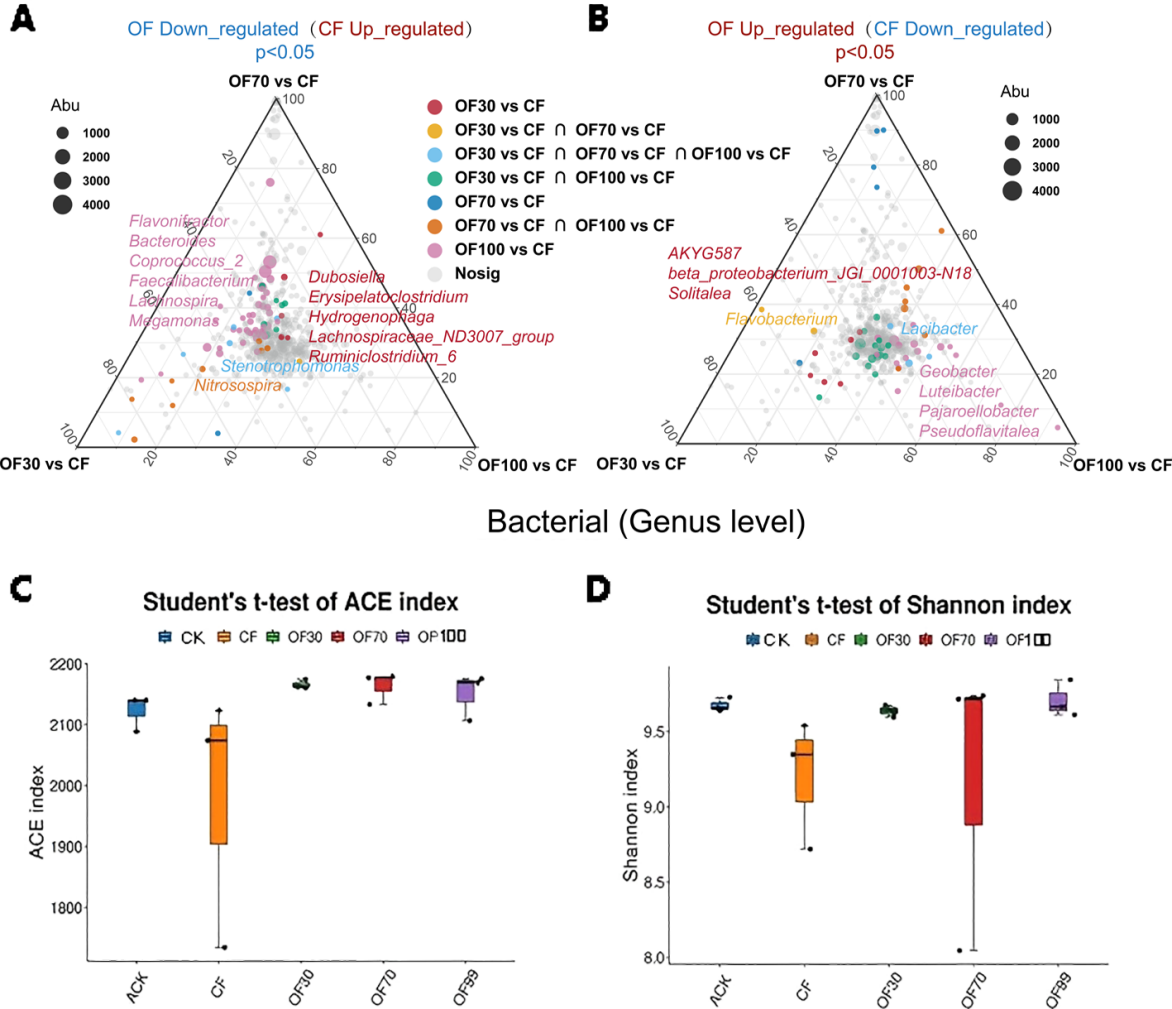
Organic-inorganic fertilizer substitution reshapes the rhizosphere bacterial community structure and enriches *Flavobacterium*. **(A)** Ternary plot showing bacterial genera significantly down-regulated in organic fertilizer (OF) treatments but up-regulated in chemical fertilizer (CF) at the genus level (*P* < 0.05). **(B)** Ternary plot showing bacterial genera significantly up-regulated in OF treatments but down-regulated in CF (*P* < 0.05). Each point represents a differentially abundant bacterial genus, and its position reflects the relative contribution of OF30 vs CF, OF70 vs CF, and OF100 vs CF. Colors indicate genera uniquely or commonly enriched among treatments, and point size represents relative abundance. **(C)** ACE index showing changes in bacterial community richness under different fertilization regimes. **(D)** Shannon index showing changes in bacterial community diversity under different fertilization regimes. Differences were considered statistically significant at *P* < 0.05.

Consistent with the improvements in soybean growth and soil fertility (**Figure 1**, **Table 2**), organic-inorganic fertilizer substitution significantly reshaped the rhizosphere bacterial community (**Figure 2**).

Genus-level ternary plot analysis revealed a clear fertilization-dependent redistribution of bacterial taxa across organic fertilizer (OF) and chemical fertilizer (CF) treatments (**Figures 2A, B**). Multiple genera exhibited significantly higher relative abundance under CF but reduced representation under OF, with data points clustering toward the CF vertex (**Figure 2A**). These taxa included *Flavonifractor*, *Bacteroides*, *Coprococcus_2*, *Faecalibacterium*, *Lachnospira*, *Megamonas*, and additional CF-associated genera such as *Dubosiella*, *Erysipelatoclostridium*, *Hydrogenophaga*, and *Ruminiclostridium_6*, indicating preferential maintenance under mineral nutrient-dominated conditions.

Conversely, a distinct subset of bacterial genera displayed significant enrichment under OF treatments while remaining comparatively underrepresented in CF soils (**Figure 2B**). Notably, *Flavobacterium*, *Lacibacter*, *Geobacter*, *Luteibacter*, *Pajaroellobacter*, *Pseudoflavitalea*, *Solitalea*, and selected *beta-Proteobacteria* consistently shifted toward OF30, OF70, and OF100 vertices, reflecting a progressive association with organic nutrient inputs. Several of these taxa are commonly linked to organic matter decomposition, redox-active metabolism, nutrient mobilization, and rhizosphere-associated carbon processing, suggesting that OF treatments support a broader spectrum of metabolically versatile bacterial groups. And FAPROTAX analysis showed that, compared with CF, OF30 and OF70 significantly increased the relative abundances of functional groups associated with nitrite respiration, anoxygenic photoautotrophy, anoxygenic photoautotrophy sulfur oxidizing, denitrification, nitrate denitrification, nitrous oxide denitrification, and nitrite denitrification. In addition, OF70 showed a significant increase in cellulolysis (**Supplementary Figure 5A**), and these functions were generally more enriched in OF70 than in OF30 (**Supplementary Figure 5B**). Beyond compositional changes, OF substitution was accompanied by significant increases in bacterial α-diversity relative to CF (**Figures 2C, D**). ACE richness index was elevated under OF30 and OF70, indicating an expansion in detectable taxonomic breadth under partial organic replacement. Similarly, the Shannon diversity index increased under OF treatments, reflecting enhanced community evenness and reduced dominance by a limited subset of taxa.

Overall, these findings suggest that organic-inorganic fertilizer substitution, particularly at moderate levels, may help promote a more diverse and metabolically broader rhizosphere bacterial community than chemical fertilization alone.

### 3.5 Rhizosphere metabolome exhibits conserved structure with targeted lipid- and carbon-class enrichment under moderate organic substitution

Untargeted metabolomic analyzes detected 1,590 metabolites from 15 samples across five treatments (**Supplementary Table 2**), denoting high chemical complexity at soybean harvest. Overall metabolite composition was similar across treatments, reflecting a conserved rhizosphere chemical landscape with treatment-dependent tuning rather than full metabolome turnover. Pairwise bacterial community analysis (**Supplementary Figure 4**) confirmed high replicate reproducibility and fertilization-driven clustering. OF30/OF70 showed the strongest within-group similarity, followed by CK/CF; OF100 had similar reproducibility with wider dispersion. High inter-group similarity supported the large shared core microbiome (**Supplementary Figure 3**).

**Figure 3:**
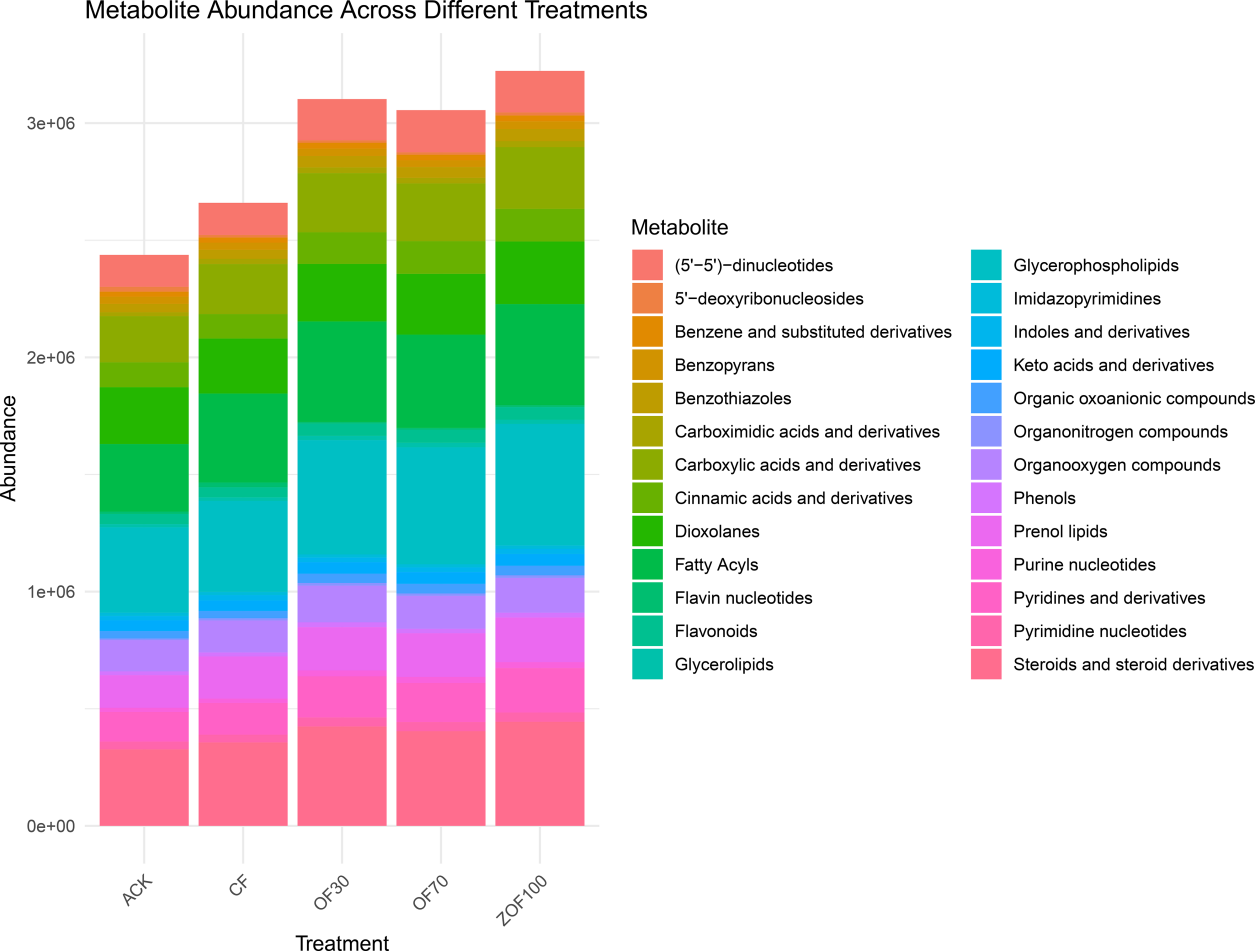
Class-level composition of rhizosphere metabolites across fertilization regimes. Stacked bar plots show the cumulative abundance of the top 26 annotated metabolite classes in rhizosphere soils under five fertilization treatments (CK, CF, OF30, OF70, and OF100) at the soybean harvest stage. Metabolites were grouped into major biochemical classes based on structural annotation, and bars represent the summed signal intensity per class across biological replicates.

At the soybean harvest, glycerophospholipids, steroids/steroid derivatives, and fatty acyls dominated metabolite profiles across all treatments, accounting for most total metabolite abundance (**Figure 3**). Along the organic substitution gradient, several directional yet moderate metabolic shifts occurred. Glycerophospholipids increased progressively from CK/CF to OF70/OF100, peaking in cumulative abundance. Steroids and fatty acyls varied by treatment but were generally more abundant under organic fertilizer substitution (30%, 70% and 100%) than CK/CF. Mid-abundance classes (carboxylic acids and derivatives, dioxolanes, prenol lipids) were stable, rising slightly under moderate organic substitution (OF30/OF70), consistent with accelerated carbon turnover and secondary metabolism. Low-abundance classes showed treatment-specific responses: benzothiazoles and certain keto-acid/oxoanion compounds were enriched in OF70, while 5’-deoxyribonucleosides peaked in the control and declined sharply in OF treatments. Flavin nucleotides and organonitrogen compounds were minor overall but more abundant in CK/CF, indicating sensitivity to fertilization regime.

Metabolite class composition remained broadly conserved across fertilization regimes, with organic substitution primarily reweighting dominant lipid- and carbon-associated metabolite pools rather than restructuring the metabolome. Organic-inorganic fertilization (OF30/OF70) was associated with the most consistent increases in carbon-related metabolite classes, whereas OF100 produced limited additional shifts.

### 3.6 Organic–inorganic fertilizer substitution reshapess rhizosphere metabolite landscapes toward functionally supportive chemical environments

**Figure 4:**
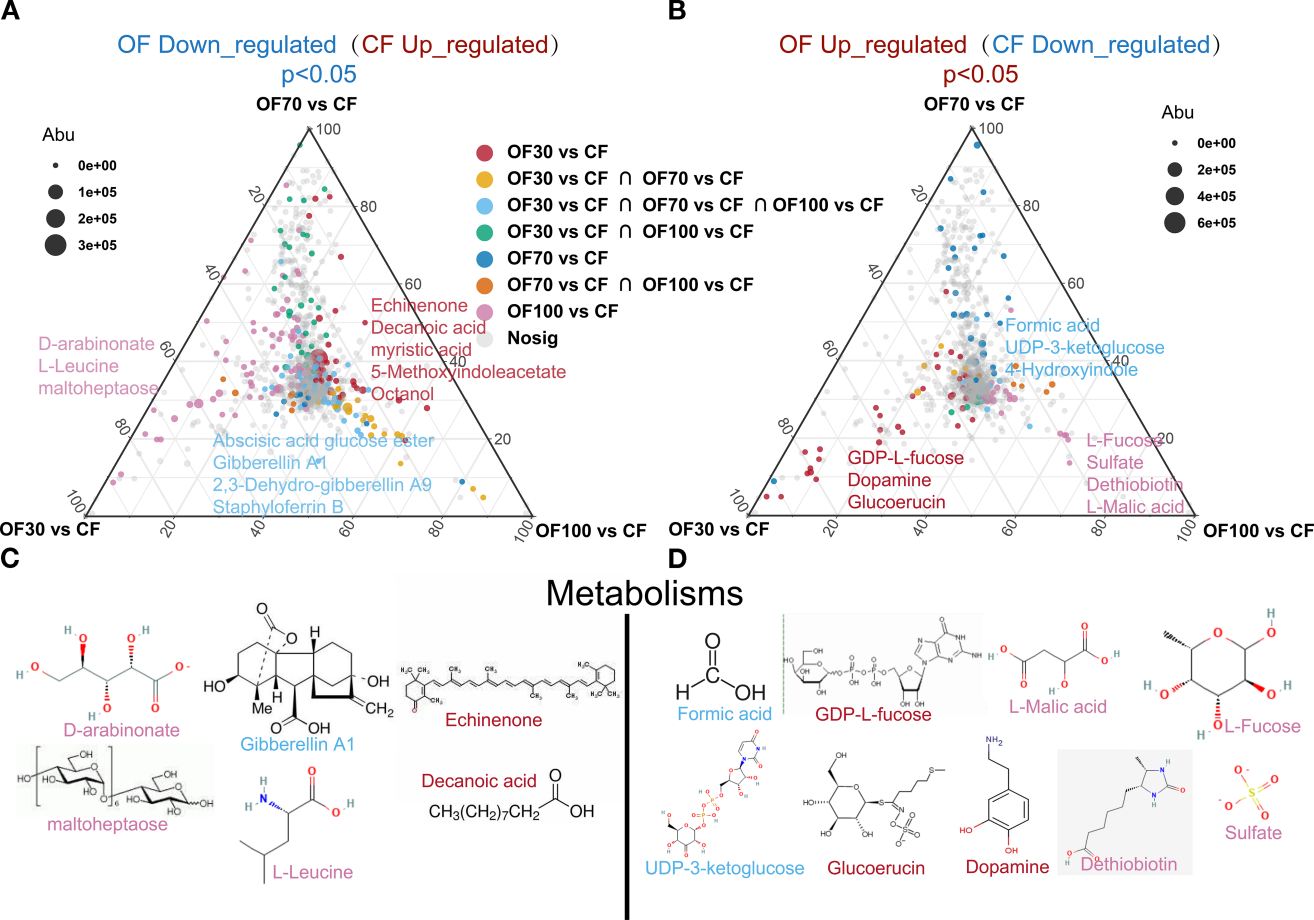
Differential rhizosphere metabolite profiles in response to organic–inorganic fertilizer substitution, Delphinidin significantly enriched in OF30 and OF70. **(A)** Ternary plot showing metabolites significantly down-regulated in organic fertilizer treatments (OF) but up-regulated in chemical fertilizer (CF) compared with CF (DESeq2, P < 0.05). **(B)** Ternary plot showing metabolites significantly up-regulated in OF treatments but down-regulated in CF (*P* < 0.05). Each point represents a differential metabolite, and its position reflects the relative contribution of OF30 vs CF, OF70 vs CF, and OF100 vs CF. Colors indicate metabolites uniquely or commonly enriched among different treatments. Point size corresponds to metabolite abundance. **(C)** Representative chemical structures of metabolites significantly reduced under OF treatments. **(D)** Representative chemical structures of metabolites significantly enriched under OF treatments, highlighting Delphinidin, which was markedly enriched in OF30 and OF70 treatments. Differences were considered statistically significant at *P* < 0.05.

Integrating rhizosphere metabolomic and soybean phenotypic profiles revealed organic-inorganic substitution reshaped rhizosphere metabolism via coordinated shifts in assimilated carbon allocation, secondary metabolism and signaling (**Figure 4**).

Ternary plot analysis revealed that several metabolites remained significantly elevated under chemical fertilization (CF) but were reduced under organic fertilizer (OF) treatments (**Figure 4A**). These compounds included lipid-derived intermediates and stress-associated metabolites such as echinenone, decanoic acid, myristic acid, and 5-methoxyindoleacetate, suggesting that CF maintained a rhizosphere chemical milieu enriched in stress-related or potentially inhibitory metabolic byproducts. In contrast, OF treatments-particularly OF30 and OF70-selectively enriched metabolites associated with carbohydrate metabolism, organic acid turnover, antioxidant capacity, and phytohormone-related pathways (**Figure 4B**). Significantly up-regulated metabolites included formic acid, UDP-3-ketoglucose, GDP-L-fucose, glucoside derivatives, dopamine, delphinidin, L-fucose, L-malic acid, sulfate, and dethiobiotin. Structural annotation further highlighted divergent chemical signatures between fertilization regimes (**Figures 4C, D**). Metabolites reduced under OF were predominantly lipid-associated and stress-linked secondary compounds, whereas metabolites enriched under OF were dominated by organic acids, sugar conjugates, phenylpropanoids, and flavonoid-type antioxidants. This expansion of bioavailable carbon substrates likely enhances microbial metabolic versatility and contributes to a chemically buffered rhizosphere environment. Among the differentially accumulated metabolites, delphinidin, a flavonoid-derived anthocyanin, exhibited particularly strong enrichment under OF30 and OF70, emerging as a responsive metabolic marker of organic fertilizer input (**Figures 4B, D**). Given the established roles of flavonoids in microbial recruitment, antioxidant buffering, Nod factor signaling, and nodule development, delphinidin accumulation provides a plausible mechanistic bridge linking rhizosphere carbon redistribution with enhanced symbiotic interactions and nitrogen fixation, consistent with observed increases in nodulation and nitrogenase activity (**Figures 1I, J**).

Collectively, these results indicate that organic fertilizer substitution reshapes the rhizosphere chemical milieu toward a metabolite landscape characterized by greater carbon-use efficiency, redox balance, and signaling potential. Rather than broadly increasing metabolite abundance, OF appears to promote a coordinated optimization of chemical resource allocation, creating conditions that favor cooperative microbial assembly, reinforce beneficial plant-microbe interactions, and support improved nutrient acquisition and productivity under intermediate substitution levels.

### 3.7 Moderate organic substitution reshapes peripheral fungal diversity while preserving a conserved core rhizosphere assemblage

High-throughput sequencing of the rhizosphere fungal communities generated 1,856,742-1,945,318 paired-end reads for the five treatments (CK, CF, OF30, OF70, and OF100). After merging and quality filtering, 1,723,456 and 1,812,945 clean tags were obtained (**Supplementary Table 3**). The observed OTU coverage ranged from 98.5% to 99.2%, indicating high sequencing completeness, which was further supported by rarefaction curves reaching clear saturation plateaus across all treatment groups (**Supplementary Figure 6**).

At the soybean harvest stage, the phylum level, *Ascomycota* was dominant across all treatments, while *Basidiomycota* and *Glomeromycota* were also relatively abundant, with their proportions varying among treatments. Other phyla, including *Mortierellomycota*, *Chytridiomycota*, *Rozellomycota*, and unclassified taxa, contributed smaller proportions (**Supplementary Figure 6**).

Community structure at the phylum level remained broadly conserved, indicating strong stability of the core fungal assemblage (**Supplementary Figure 7**). Nevertheless, moderate organic substitution (OF30-OF70) produced consistent but modest directional shifts. Relative to CK and CF, Mortierellomycota and other saprotrophic lineages showed higher proportional abundance under OF30 and OF70, whereas Basidiomycota declined under CF and partially recovered under organic inputs. Ascomycota remained dominant but exhibited slight proportional reductions under OF30/OF70 as other functional groups expanded. At the highest substitution level (OF100), phylum-level composition largely resembled OF70 with slightly greater dispersion among minor taxa, indicating no major restructuring.

Venn diagram further revealed a substantial shared fungal core, with 390 OTUs common to all treatments (**Supplementary Figure 8**), confirming high conservation of dominant fungal taxa. Total richness varied modestly among regimes (616–797 OTUs), peaking under OF30 and OF70, which exceeded CK and CF, whereas OF100 showed no further increase and slightly reduced richness. Treatment-specific OTUs were limited but more evident under moderate organic substitution (OF30/OF70), suggesting expansion of low-abundance peripheral taxa rather than turnover of dominant groups.

Organic fertilizer substitution primarily reweights fungal functional guilds rather than restructuring the core community. Organic-inorganic fertilization (OF30/OF70) enhances saprotrophic and carbon-cycling taxa (e.g., Mortierellomycota) and increases fungal richness, while full substitution (OF100) leads to diminishing returns. This indicates that intermediate organic inputs can enhance diversity and functional diversification while preserving a stable core rhizosphere fungal microbiome.

### 3.8 Moderate organic substitution reshapes rhizosphere fungal assembly towards carbon-cycling and plant-beneficial guilds

**Figure 5:**
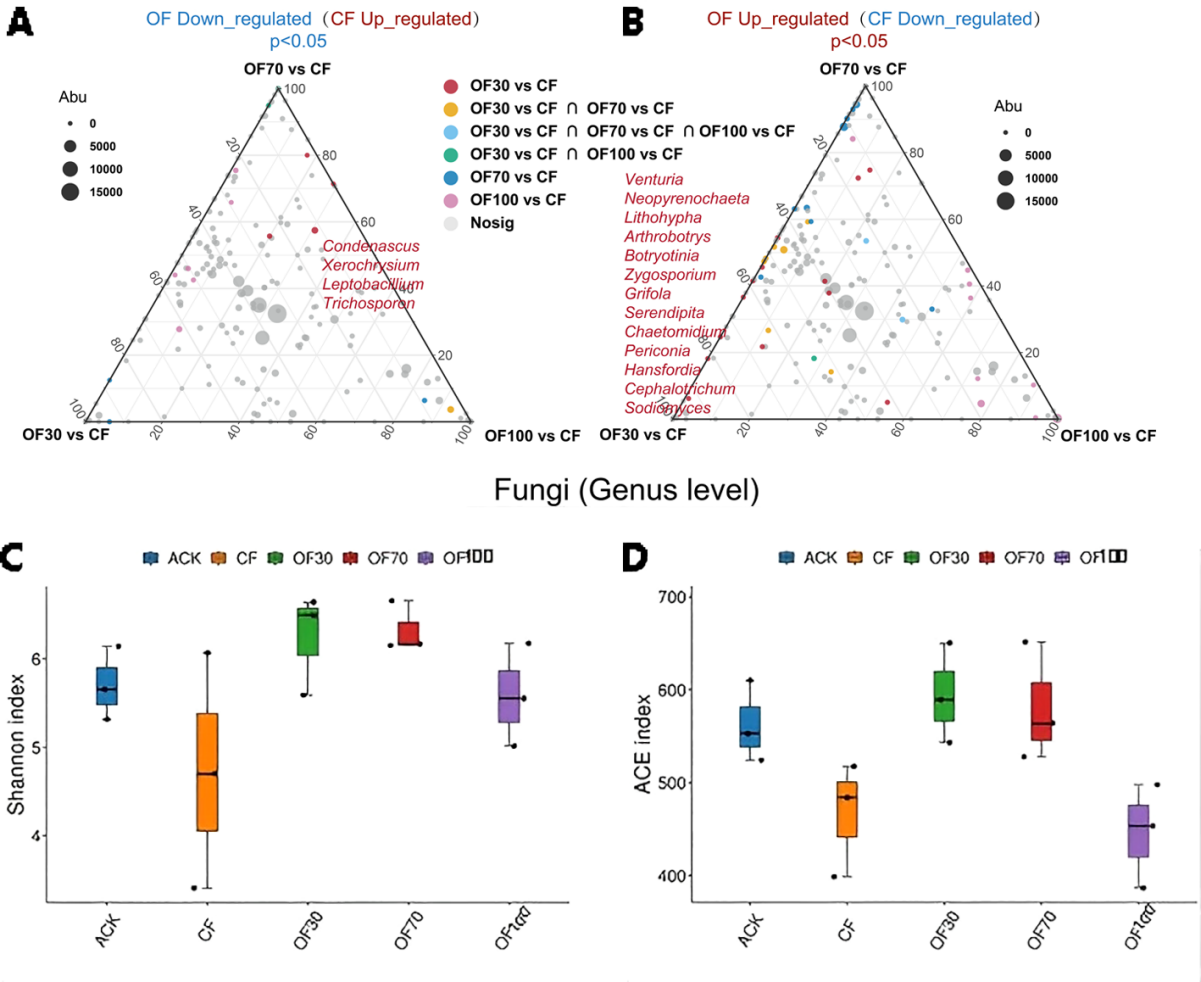
Moderate organic fertilizer substitution increases rhizosphere fungal α-diversity and reshapes fungal community structure. **(A)** Ternary plot showing fungal genera significantly down-regulated in organic fertilizer (OF) treatments but up-regulated in chemical fertilizer (CF) at the genus level (*P* < 0.05). **(B)** Ternary plot showing fungal genera significantly up-regulated in OF treatments but down-regulated in CF (*P* < 0.05). Each point represents a differentially abundant fungal genus, and its position reflects the relative contribution of OF30 vs CF, OF70 vs CF, and OF100 vs CF. Point size indicates relative abundance. **(C)** Shannon index of rhizosphere fungal communities under different fertilization treatments. **(D)** ACE index of rhizosphere fungal communities under different fertilization treatments. Differences among treatments were considered statistically significant at *P* < 0.05.

Given coordinated shifts in soil nutrients, bacterial composition, and metabolite pools (**Figures 1**-**4**), we next examined fungal community responses to organic fertilizer substitution (**Figure 5**). Organic inputs induced a clear but selective restructuring of fungal communities, consistent with carbon-driven niche filtering rather than wholesale taxonomic turnover.

Ternary analyzes revealed that several fungal genera enriched under chemical fertilization were reduced under organic regimes, whereas a distinct cohort of taxa was preferentially associated with OF treatments (**Figures 5A, B**). Genera such as *Trichosporon*, *Xeromyces*, and *Leptobacillium* showed higher relative contributions under CF (**Figure 5A**), whereas OF30 and OF70 favored enrichment of saprotrophic and plant-associated genera including *Serendipita*, *Chaetomium*, *Arthrobotrys*, *Grifola*, *Peniophora*, *Zygosporium*, *Hansfordia*, and *Cephalotrichum* (**Figure 5B**). Many of these taxa are functionally associated with lignocellulose degradation, organic matter mineralization, nutrient mobilization, pathogen suppression, and plant growth promotion, indicating a transition toward carbon-efficient and rhizosphere-beneficial fungal guilds under moderate organic fertilizer input. Fungal α-diversity increased under OF30 and OF70, as indicated by higher Shannon and ACE indices (**Figures 5C, D**). In contrast, OF100 did not further enhance diversity, suggesting that moderate organic inputs maximize niche heterogeneity while preventing carbon-driven competitive exclusion.

Importantly, fungal restructuring paralleled bacterial and metabolite dynamics observed in **Figures 2**-**4**. Elevated pools of flavonoids, organic acids, and carbohydrate derivatives likely expanded substrate niches for saprotrophic fungi, while enrichment of *Flavobacterium* may further modulate fungal recruitment through cross-kingdom metabolic signaling and competitive interactions.

### 3.9 Cross-kingdom interaction networks optimize soybean productivity

To resolve cross-domain coordination under moderate organic substitution (OF30/OF70), an association network showed that growth tightly links yield traits, with positive correlations among plant height, root length, biomass, photosynthesis, and seed weight. SOC, AP, and AK were positively associated with these traits. Flavonoids, phenylpropanoids, and organic acid derivatives were most strongly associated with nitrogen fixation and photosynthesis (**Supplementary Figure 9**).

**Figure 6:**
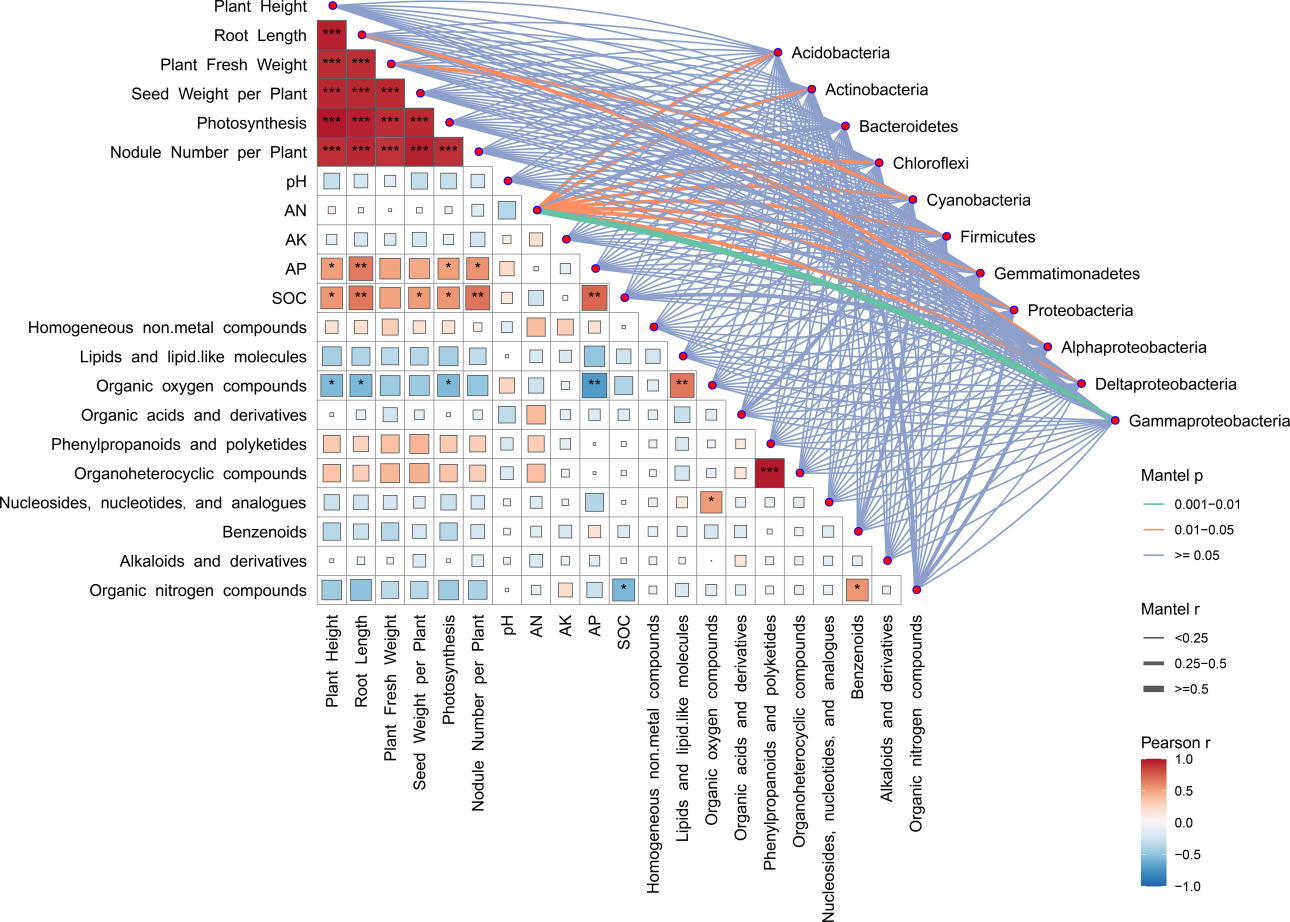
Integrated correlation–Mantel network linking plant traits, soil properties, metabolite classes, and bacterial phyla. The lower triangular matrix depicts pairwise Pearson correlations among soybean agronomic traits, soil physicochemical variables (pH, AN, AP, AK, SOC), and metabolite classes. Color intensity represents the correlation coefficient (r), with red indicating positive correlations and blue indicating negative correlations. Squares denote significant associations (*P* < 0.05). The overlaid network on the right illustrates Mantel test–based associations between bacterial phyla and the combined plant–soil–metabolite matrix. Edge color indicates statistical significance, and line thickness corresponds to Mantel’s r value.

**Figure 7:**
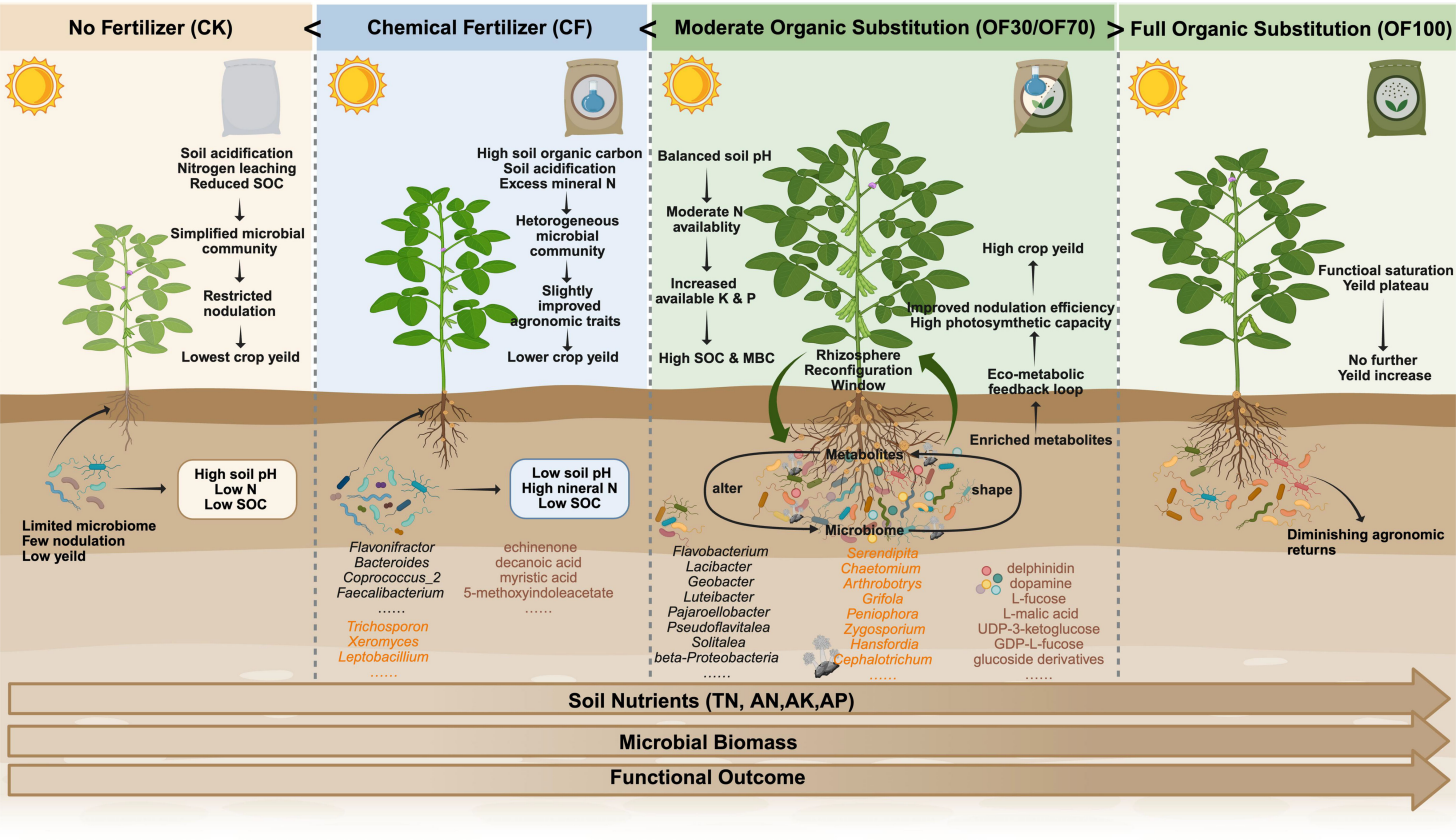
Organic fertilizer substitution reshapes rhizosphere biogeochemistry, microbiome assembly, and functional outcomes along a management gradient. Conceptual model illustrating rhizosphere responses under four fertilization regimes: no fertilizer (CK), chemical fertilizer (CF), moderate organic substitution (OF30/OF70), and full organic substitution (OF100). CK is characterized by nutrient limitation, reduced microbial diversity, restricted nodulation, and low yield. CF increases mineral N availability but induces soil acidification and SOC depletion, leading to simplified microbial communities and constrained symbiotic efficiency despite elevated inorganic inputs. Moderate organic substitution establishes a balanced soil pH and nutrient profile (TN, AN, AK, AP), enhances SOC and microbial biomass carbon (MBC), and promotes a reconfigured rhizosphere interaction network. This regime enriches beneficial bacterial and fungal taxa and carbon-associated metabolites (e.g., flavonoids, glycerophospholipids, organic acids), forming a tightly coupled microbe–metabolite module that enhances nodulation efficiency, photosynthetic capacity, and crop yield. The central circular module denotes a bidirectional eco-metabolic feedback loop in which microbes reshape rhizosphere metabolites, and metabolites reciprocally select and sustain microbial assemblages, thereby reinforcing rhizosphere function under moderate organic substitution. In contrast, full organic substitution (OF100) leads to functional saturation, where additional organic inputs do not further increase yield, indicating diminishing agronomic returns. Arrows at the bottom depict trends across treatments in soil nutrients, microbial biomass, and functional outputs, highlighting moderate organic substitution as an optimal balance point for coordinated rhizosphere reprogramming and soybean productivity.

Based on the correlation framework, an integrative cross-domain network resolves potential interactions among taxonomic groups (**Supplementary Table 4**, **Figure 6**). SOC displayed the broadest positive associations, strongly correlating with AP and with key growth and yield traits, including nodule number per plant, root length, plant height, photosynthesis, and seed weight per plant, while negatively correlating with organic nitrogen compounds. AP, beyond its tight linkage with SOC, was also positively associated with root length, nodule number per plant, plant height, and photosynthesis. At the metabolite level, organic oxygen compounds showed predominantly negative associations, being strongly negatively correlated with AP and with root length, plant height, and photosynthesis. In contrast, they were positively associated with lipids and lipid-like molecules and with nucleosides, nucleotides, and their analogues. Organic nitrogen compounds were negatively linked to SOC but positively correlated with benzenoids. Mantel analysis further revealed positive correlations between AN and multiple bacterial phyla, including *Acidobacteria*, *Actinobacteria*, *Chloroflexi*, *Cyanobacteria*, *Firmicutes*, *Gemmatimonadetes*, and *Proteobacteria*. Within *Proteobacteria*, *Deltaproteobacteria* showed moderate associations, whereas *Gammaproteobacteria* exhibited strong positive correlations with AN. *Cyanobacteria* were positively associated with plant fresh weight, and Alphaproteobacteria with root length. And **Figure 7** summarizes that organic-inorganic fertilization (OF30/OF70) was associated with coordinated improvements in rhizosphere nutrient status, microbial community structure, metabolite accumulation, nodulation, and soybean yield compared with no fertilizer, chemical fertilizer, or full organic substitution treatments.

## 4 Discussion

### 4.1 Moderate organic fertilizer substitution supports soybean productivity through coordinated physiological regulation

Moderate organic fertilizer substitution (OF30/OF70) consistently outperformed both exclusive chemical fertilization (CF) and full organic replacement (OF100) across vegetative growth, nodulation, photosynthetic performance, and yield-related traits (**Figure 1**). This non-linear productivity response indicates that crop performance is constrained not by absolute nutrient abundance, but by the alignment between nutrient availability, carbon supply, and plant metabolic demand (Zhao et al., 2020; Wang et al., 2024a). Such a pattern aligns with recent evidence that nutrient balance, rather than nutrient saturation, contributes to crop performance in intensive agricultural systems (Wu et al., 2025).

Rather than promoting nutrient balance, moderate organic inputs appear to establish a homeostatic nutrient regime (Wang et al., 2020; Lin et al., 2021; Vendig et al., 2023; Wu et al., 2023). The pronounced enhancement of nodulation and nitrogenase activity under OF30-OF70 further suggests that partial organic substitution alleviates mineral-N-mediated repression of nodulation signaling and root nitrogen sensing pathways (**Figures 1I, J**). Higher inorganic nitrogen has been shown to suppress nod gene expression and disrupt root-rhizosphere signaling networks (Wu et al., 2024b, c), whereas organic amendments can temper mineral N exposure while maintaining sufficient nitrogen supply (Ma et al., 2023b).

Importantly, yield gains under OF30/OF70 (**Figures 1L, M**) reflect improved coordination across carbon fixation, nitrogen metabolism, and biomass allocation, rather than simple nutrient enrichment. This finding advances a conceptual shift away from fertilizer intensity toward nutrient and carbon synchrony as a primary determinant of crop productivity, highlighting the potential of balanced fertilization strategies to optimize physiological efficiency rather than maximize input load.

### 4.2 Soil carbon enrichment and nutrient rebalancing act as upstream of rhizosphere function

The substantial improvements in soybean performance are intrinsically linked to the modulated rhizosphere soil fertility (Ren et al., 2025). Organic fertilizer substitution induced coordinated restructuring of rhizosphere soil chemistry, including pH buffering, soil organic carbon (SOC) enrichment, and redistribution of nitrogen (N), phosphorus (P), and potassium (K) pools (Ren et al., 2021; Wang et al., 2026). Among these changes, SOC, AP, AK, and pH all act as important upstream regulators, consistent with **Supplementary Figure 9**. This aligns with the core consensus of current agricultural soil carbon research, which identifies SOC enrichment as a key regulatory node linking fertilization management to rhizosphere ecological function and crop productivity (Bai et al., 2024).

Intermediate substitution levels (OF30/OF70) sustained adequate plant-available nitrogen while avoiding excessively high mineral nitrogen (**Table 2**), a condition frequently associated with nitrate leaching, soil acidification, and microbial community simplification (Sun et al., 2021; Chen et al., 2022; Zhang et al., 2022). This nutrient rebalancing dampens temporal volatility in mineral nutrient pools, contributing to a more chemically buffered and resilient soil environment (Tang et al., 2023; Luo et al., 2024). Notably, the soil state generated under OF30/OF70 differs functionally from both CF (mineral-N-saturated, carbon-limited) and OF100 (carbon-rich but potentially nutrient-constrained). It suggests that productive rhizosphere function emerges from balanced carbon–nutrient coupling rather than extremes of either input type (Nosengo, 2003; Dunn and Gilbertson, 2025; Wang et al., 2025).

By simultaneously enhancing carbon availability and moderating nutrient fluctuations, organic-inorganic fertilization establishes a resource-efficient soil regime capable of sustaining microbial activity, nutrient turnover, and long-term fertility (Huang et al., 2025). These findings reinforce the broader principle that soil carbon management represents an important regulatory factor influencing rhizosphere biological processes and crop productivity (Han et al., 2025; Luo et al., 2025).

### 4.3 Organic-inorganic fertilization promotes directional bacterial turnover and expands functional redundancy

A shared bacterial core persists across organic fertilization (**Supplementary Figure 3**), demonstrating microbial resilience through phylum-level stability and functional redundancy. Organic inputs fine-tune rather than disrupt community assembly, preserving beneficial consortia for nutrient cycling and disease suppression. This buffering capacity can inform biofertilizer designs that leverage core microbiomes for climate-resilient agriculture. And organic-inorganic fertilization drove turnover in rhizosphere bacterial communities, selectively enriching metabolically versatile and carbon-responsive taxa such as *Flavobacterium*, *Geobacter*, *Luteibacter*, and *Pseudoflavitalea* (**Figure 2**). These genera are widely associated with organic matter decomposition, redox cycling, polysaccharide utilization, and nutrient mobilization, indicating a transition toward microbial assemblages optimized for processing complex carbon substrates rather than rapidly available mineral nutrients.

Beyond compositional shifts, increases in bacterial richness and evenness under OF30/OF70 suggest an expansion of functional redundancy, a recognized stabilizing property of microbial ecosystems that buffers nutrient cycling against environmental variability and disturbance. Reduced dominance by mineral-adapted taxa likely enhances metabolic flexibility, enabling microbial communities to sustain nutrient turnover across fluctuating soil resource conditions (Huang et al., 2021; Ma et al., 2023a).

This diversification is consistent with ecological theory predicting that carbon-enriched environments support more diverse, functionally resilient microbial networks, which in turn promote more stable nutrient provisioning to plants (Pan et al., 2025). Collectively, these patterns provide mechanistic support for the hypothesis that organic fertilizer fosters microbiome configurations that enhance both ecosystem resilience and agronomic performance through increased functional breadth and redundancy (Chen et al., 2025; Sun et al., 2025).

### 4.4 Rhizosphere metabolome reprogramming links carbon allocation to microbial recruitment and plant performance

Moderate organic substitution (30/70%) preserves the rhizosphere metabolome’s core while enriching lipid/carbon metabolites (glycerophospholipids, steroids, fatty acyls) and carboxylic acids, signaling increased carbon availability for microbial and plant functions (**Figure 3**). OF70-specific benzothiazoles and keto-acids reveal targeted pathway activation. Organic amendments thus amplify carbon accessibility, stabilizing rhizosphere productivity through metabolic refinement rather than structural disruption.

This shift indicates a transition of the nutrient-imbalanced chemical state to a metabolically supportive, signal-rich ecological niche. The substantial accumulation of compounds like formic acid, UDP-3-ketoglucose, and particularly delphinidin, a flavonoid, emerged as a prominent OF-responsive compound (**Figure 4B**), consistent with known roles of flavonoids in redox buffering, modulation of root oxidative status, and microbial recruitment (Hodnick et al., 1988; Shen et al., 2022). It provides a direct mechanistic link between metabolite changes and the observed improvements in nodulation and nitrogen fixation. This metabolic reprogramming may enhances rhizosphere microecological functions, and promotes nodule maturation by regulating key nodulation-related genes. Delphinidin-mediated signaling likely fine-tunes symbiotic recognition timing, while accelerated organic acid turnover improves iron/molybdenum bioavailability synergistically stabilizing nitrogenase activity to support nitrogen fixation (Wang et al., 2024b).

Although direct causal validation remains necessary, the metabolite patterns observed here support a model in which organic fertilizer redirects rhizosphere carbon flux toward bioactive and signaling-rich metabolite pools, thereby shaping microbial niche selection and reinforcing plant physiological resilience (Xun et al., 2021; Qiao et al., 2025). More broadly, these findings highlight metabolite-mediated niche structuring as a biochemical interface linking soil carbon inputs to microbiome assembly and plant performance (Xie et al., 2022). This positions the rhizosphere metabolome not merely as a downstream product of microbial activity, but as an active regulatory layer capable of modulating cross-kingdom interactions and functional outcomes (Jacoby and Kopriva, 2019; Wang et al., 2024c; Pantigoso et al., 2025).

### 4.5 Cross-kingdom microbiome restructuring suggests organic fertilizer acts as an ecological niche filter

The fungal community transition from oligotrophic Basidiomycota (CF) to copiotrophic Mortierellomycota (OF) (**Supplementary Figure 7**) reflects fundamental carbon use strategy shifts. Beyond bacterial responses, organic-inorganic fertilization coordinately reshaped fungal community assembly, selectively enriching saprotrophic, nutrient-mobilizing, and plant-associated taxa, including *Serendipita*, *Chaetomium*, and *Arthrobotrys* (**Figure 5**). These genera are functionally linked to lignocellulose degradation, organic matter mineralization, pathogen suppression, and root-zone functional support, indicating a shift toward fungal guilds adapted to carbon-rich and biologically active soil niches (Li et al., 2017; Hannula and Träger, 2020; Liu et al., 2023a). *Serendipita’s* promotion, linked to mycorrhizal-like symbioses, likely bolsters P uptake and drought tolerance, synergizing with AP elevations (**Table 2**). *Arthrobotrys* enrichment implies nematode biocontrol, addressing a major soybean constraint. Diversity surges (**Figure 5D**) at moderate OF reflect niche expansion via metabolite cues like flavonoids (**Figure 4**), fostering cross-kingdom synergies with bacteria (e.g., *Flavobacterium*-fungal *antagonism*). This guild reorientation, without disrupting the core community structure, is consistent with ecological filtering under moderate organic substitution and may contribute to improved rhizosphere functioning and soybean performance.

Our findings suggest that moderate organic-inorganic mixing can reduce chemical fertilizer input by 70% while maintaining or increasing yield, making it a practical strategy for sustainable soybean production. It may aligns with the core direction of China’s agricultural greenhouse gas emission reduction and sustainable agricultural development, as it achieves the dual goals of reducing chemical fertilizer input and maintaining crop productivity, while also improving soil health and reducing agricultural non-point source pollution (Wu et al., 2024a).

Previous studies demonstrated that chemical nitrogen excess shifts the soybean rhizosphere microbiome toward mineral-nutrient dependency, impairing nodulation signaling and redox equilibrium. Organic management activates a carbon-gating mechanism, reorganizing microbial activity from basic mineralization to symbiosis support and systemic resistance induction (Chen et al., 2023). This transition replaces energy-costly industrial nitrogen fixation with photosynthetic-powered biological fixation, while establishing self-sustaining rhizosphere networks (Cheng et al., 2022). Continuous SOC renewal imposes niche selection on microbial guilds, whose organic matter processing reciprocally sustains soil health, forming a closed-loop system. And optimizing soil microbial functions can enhance nutrient acquisition, thereby improving nutrient availability and crop productivity (Zhang et al., 2026a). Collectively, **Supplementary Figure 8** delineates the statistical coupling architecture across domains, whereas **Figure 6** resolves the potential ecological pathways underlying this coupling at the taxonomic level. We further propose a mechanistic framework in which moderate organic inputs prepromote a more balanced, carbon-enriched, and biologically active rhizosphere, ultimately may enhancing soybean performance (**Figure 7**), offering a basis for refining fertilization strategies in soybean and potentially other cropping systems. Nevertheless, these findings should be interpreted within the boundaries of the present experimental system. Although the present study demonstrates the advantage of moderate organic substitution under a controlled greenhouse pot system, its broader applicability across soybean production systems should be interpreted with caution, as responses may vary with soil type, cultivar, and environmental conditions. Further multi-environment field validation will be needed to confirm its generalizability.

## 5 Limitation of the study

This work provides a mechanistic blueprint for microbiome-informed fertilization strategies, supporting the development of resilient and resource-efficient legume-based systems. While this study illuminates organic substitution mechanisms, key questions persist, (1) How do initial soil carbon stocks modulate substitution efficacy? (2) What is the role of viral communities in reshaped microbiomes? (3) Can metabolite signatures predict optimal substitution rates across soil types? Moreover, while correlative patterns support the proposed causal chain, experimental manipulation, isotope tracing, and functional genomics will be required to confirm direct mechanistic linkages among soil chemistry, metabolites, microbial taxa, and plant traits. And future field studies should include economic cost-benefit analysis to improve farmer acceptance.

## 6 Conclusion

This study demonstrate coordinated non-linear optimization across soil chemistry, microbiome diversity, metabolite pools, and soybean yield under nutrient-equalized substitution gradient. Moderate organic inputs promote a carbon-enriched, chemically buffered, and biologically active rhizosphere state that supports efficient nutrient uptake, stable microbial community structure, and improved plant growth. This study advances a framework in which fertilization strategies operate as ecosystem-level interventions, reshaping rhizosphere causal networks rather than functioning as isolated nutrient delivery tools. It highlights carbon-driven microbiome assembly and metabolite-mediated niche filtering as key mechanisms linking soil management to crop performance, may providing a mechanistic foundation for predictive fertilization models that integrate soil chemistry, microbiome dynamics, and plant physiology. Nevertheless, because this study was performed under a controlled greenhouse pot system with one soil type and one soybean cultivar, the broader applicability of the results should be interpreted cautiously. Further validation across diverse soils, cultivars, and field environments will be required to assess their generalizability.

## Data Availability

The raw sequencing data are available in the National Center for Biotechnology Information Sequence Read Archive under accession PRJNA1453983.
