# Correction: Moderate organic–inorganic fertilization optimizes soybean productivity by reshaping rhizosphere microbiome–metabolite networks

**DOI:** 10.3389/fpls.2026.1902091

**Published:** 2026-07-31

**Authors:** Jing Zhang, Qiang Liu, Jiali Chen, Yongmei Zhou, Bianhong Zhang, Zhaonian Yuan, Peiwu Li, Ziqin Pang

**Affiliations:** 1 Xianghu Laboratory, Hangzhou, China; 2 College of Natural Resources and Environment, Northwest Agriculture and Forestry University, Yangling, Shaanxi, China; 3 Fujian Key Laboratory of Agroecological Processing and Safety Monitoring, Fujian Agriculture and Forestry University, College of Agriculture, Fuzhou, China; 4 National Engineering Research Center for Sugarcane, Fujian Agriculture and Forestry University, Fuzhou, China

**Keywords:** metabolome reprogramming, organic-inorganic fertilization, rhizosphere microbiome, soil health, soybean, sustainable fertilization, symbiotic interactions, yield optimization

There was a mistake in **Figure 1G** as published. Panel G was incorrectly generated. Specifically, when preparing the figure in GraphPad Prism, the wrong set of raw data (panel C) was mistakenly copied and pasted into the panel G dataset. As a result, the bar graph in panel G became identical to panel C.

There was a mistake in **Figure 1A** as published. Due to insufficient manual proofreading during figure preparation, **Figure 1A** contained spelling mistakes and graphical inconsistencies, that have been revised as described below:

**Figure 1:**
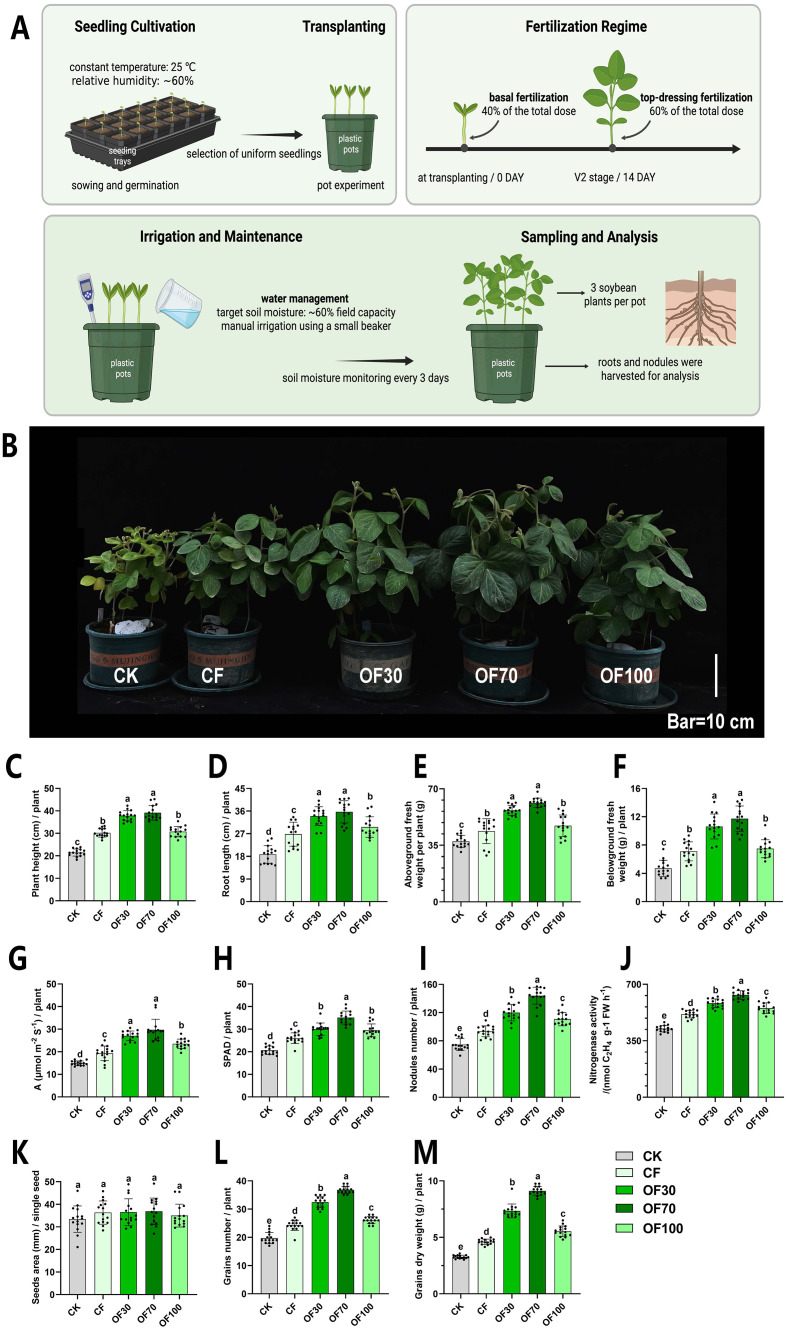
Organic–inorganic fertilizer substitution enhances soybean growth, nodulation, photosynthetic performance, and yield formation. **(A)** Schematic illustration of the soybean pot cultivation system, including seedling culture, transplanting, split fertilization, moisture management, and sampling procedures. **(B)** Phenotypic performance of soybean plants at the anthesis stage under different fertilization treatments (scale bar = 10 cm); **(C)** Plant height per plant; **(D)** Root length per plant; **(E)** Aboveground fresh weight per plant; **(F)** Belowground fresh weight per plant; **(G)** Net photosynthetic rate **(A)**; **(H)** Leaf chlorophyll content (SPAD value); **(I)** Nodule number per plant; **(J)** Nitrogenase activity per gram of fresh nodule weight; **(K)** Surface area of a single dry seed at harvest; **(L)** Total seeds number per plant at harvest; **(M)** Total dry seeds weight per plant at harvest. Different lowercase letters indicate significant differences among treatments (*P* < 0.05). CK, no fertilizer; CF, chemical fertilizer only (N–P_2_O_5_–K_2_O); OF30 and OF70, 30% and 70% substitution of inorganic fertilizer with organic fertilizer, respectively; OF100, 100% organic fertilizer derived from chicken manure and humic acid.

1. The section title “Seeding Culture” was revised to “Seedling Cultivation” to more accurately describe the seedling preparation step.

2. The environmental condition labels were standardized from “constant temlerature of 25 °C” and “relative humidity of ~60%” to “constant temperature: 25 °C” and “relative humidity: ~60%” for clearer and more consistent scientific presentation.

3. The phrase “sown and germinated” was revised to “sowing and germination” to improve grammatical consistency.

4. The phrase “selection of uniform seedings” was corrected to “selection of uniform seedlings,” thereby correcting the terminology for transplanted plant materials.

5. The transplanting-related container labels were revised from “splastic cultivation buckets” to “plastic pots,” which is clearer and more appropriate for the pot experiment.

6. The description “pot setup assays” was revised to “pot experiment” which more accurately reflects the experimental setup.

7. The section title “Fertilization Strategy” was revised to “Fertilization Regime” to better match agronomic terminology.

8. The labels “basal fertilizer” and “top-dressing fertilizer” were revised to “basal fertilization” and “top-dressing fertilization,” respectively, to describe the fertilization operations rather than the fertilizer materials themselves.

9. The time labels in the fertilization panel were revised from “Transplanting 0 days” and “14 days after transplanting” to “at transplanting/0 DAY” and “V2 stage/14 DAY,” thereby clarifying both the developmental stage and the corresponding sampling/fertilization time point.

10. The section title “Maintenance and Irrigation” was revised to “Irrigation and Maintenance” to better reflect the main experimental operation shown in this panel.

11. The watering description was revised from “artificial watering (small beaker irrigation)” to “manual irrigation using a small beaker,” which is clearer and grammatically more appropriate.

12. The soil moisture description was revised and repositioned as “soil moisture monitoring every 3 days,” improving the readability and logic of the irrigation-management workflow. “And target ~60% of field capacity” was revised to “target soil moisture: ~60% field capacity”.

13. In the sampling panel, the plant and root illustrations were replaced with clearer and more scientifically consistent graphical elements to better show the experimental sampling process.

14. The phrase “roots and nodules harvested for analysis” was revised to “roots and nodules were harvested for analysis,” improving grammatical accuracy.

15. The overall layout, alignment, arrows, font size, and graphical consistency of Figure 1 were improved to make the experimental workflow easier to follow.

No generative AI tools were used to prepare, generate, or modify **Figure 1A**. The figure was manually created and assembled by the authors using BioRender. The appropriate BioRender Publication License has been obtained.

The corrected **Figure 1** appears below.

There was a mistake in the caption of **Figure 1** as published. “(F) Belowground fresh weight per plant height” should be “(F) Belowground fresh weight per plant”. “(I) Nodules number per plant” should be “(I) Nodule number per plant”. "OF30 and OF70%, 30% and 70% substitution of inorganic fertilizer substitution with organic fertilizer, respectively" should be "OF30 and OF70, 30% and 70% substitution of inorganic fertilizer with organic fertilizer, respectively”.

The corrected caption of **Figure 1** appears below.

The original version of this article has been updated.

