# Supplementary material for: Moderate organic–inorganic fertilization optimizes soybean productivity by reshaping rhizosphere microbiome–metabolite networks
Source: Front Plant Sci. 2026 Jun 1;17:1823609. doi: 10.3389/fpls.2026.1823609 (PMC13265277; doi:10.3389/fpls.2026.1823609)
Supplement: Supplementary file 2 [file DataSheet1.docx]

**Figure S1. Rarefaction curves of rhizosphere bacterial communities under different fertilization regimes.** Rarefaction curves were generated based on the number of observed features as a function of sequencing depth for each treatment group (CK, CF, OF30, OF70, and OF100). Shaded areas indicate standard error across biological replicates. All curves approached clear saturation plateaus, indicating sufficient sequencing depth to capture the majority of bacterial diversity across samples and supporting high sequencing completeness.

**Figure S2. Phylum-level composition of rhizosphere bacterial communities across fertilization treatments.** Stacked bar plots show the relative abundance of dominant bacterial phyla in rhizosphere soils under different fertilization regimes (ACK, CF, OF30, OF70, and OF100). Only phyla with a mean relative abundance >1% across samples are displayed; remaining low-abundance taxa are grouped as “Others.” Relative abundances were calculated based on OTU counts clustered at 97% sequence similarity.

**Figure S3. Overlap of bacterial OTUs across fertilization treatments.** Venn diagram showing the overlap and uniqueness of bacterial operational taxonomic units (OTUs; clustered at 97% sequence similarity) detected in rhizosphere soils under five fertilization regimes (CK, CF, OF30, OF70 and OF100). Numbers within each sector indicate the OTUs shared among specific combinations of treatments, while numbers in non-overlapping regions represent treatment-specific OTUs. Bar plots below the Venn diagram depict the total number of OTUs detected per treatment.

**Figure S4. Pairwise similarity of rhizosphere bacterial community composition across fertilization regimes.** Heatmap showing pairwise Bray–Curtis similarity coefficients among rhizosphere bacterial communities across five fertilization treatments (CK, CF, OF30, OF70, and OF100) at the soybean harvest stage. Each cell represents the similarity between two samples, with darker blue indicating higher compositional similarity.Labels denote biological replicates (e.g., CK-1–3, CF-1–3, OF30-1–3, OF70-1–3, OF100-1–3).


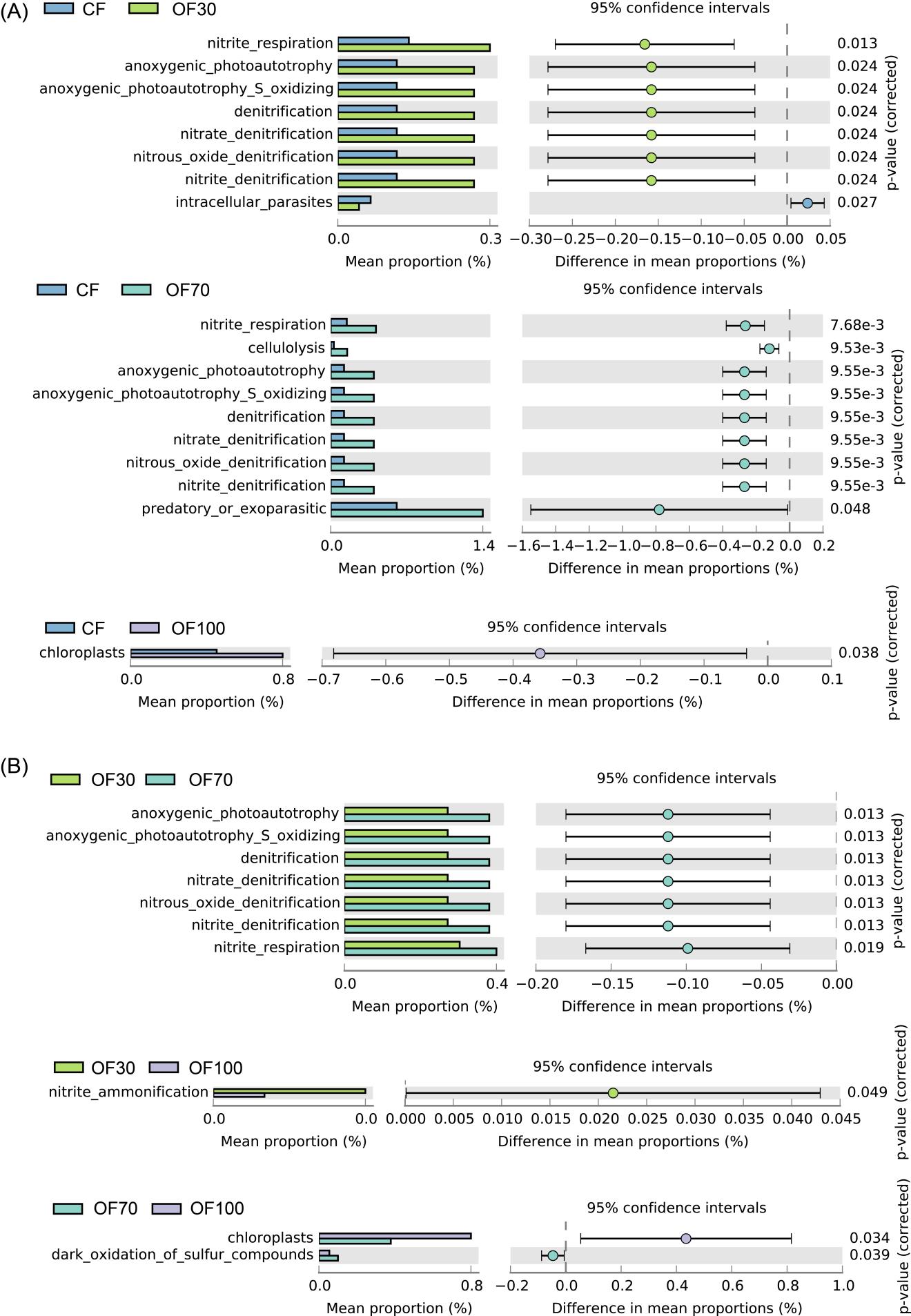


**Figure S5. FAPROTAX-predicted functional shifts in rhizosphere bacterial communities under different fertilization regimes.**

1. Differentially abundant functional groups in pairwise comparisons between CF and organic substitution treatments (OF30, OF70, and OF100). OF30 and OF70 significantly enriched several functions associated with nitrogen cycling and photoautotrophy, including nitrite respiration, anoxygenic photoautotrophy, anoxygenic photoautotrophy sulfur oxidizing, denitrification, nitrate denitrification, nitrous oxide denitrification, and nitrite denitrification. (B) Differentially abundant functional groups among OF30, OF70, and OF100. OF70 showed stronger enrichment of nitrogen-cycling and photoautotrophy-related functions than OF30, whereas OF100 differed from OF30 and OF70 in selected functions, including nitrite ammonification, chloroplasts, and dark oxidation of sulfur compounds. Bars indicate mean proportions, and points with horizontal lines indicate differences in mean proportions with 95% confidence intervals. Corrected P values are provided for each comparison.

**Figure S6. Rarefaction curves of rhizosphere fungi communities under different fertilization regimes.** Rarefaction curves were generated based on the number of observed features as a function of sequencing depth for each treatment group (CK, CF, OF30, OF70, and OF100). Shaded areas indicate standard error across biological replicates. All curves approached clear saturation plateaus, indicating sufficient sequencing depth to capture the majority of fungi diversity across samples and supporting high sequencing completeness.

**Figure S7. Phylum-level composition of rhizosphere fungi communities across fertilization treatments.** Stacked bar plots show the relative abundance of dominant bacterial phyla in rhizosphere soils under different fertilization regimes (CK, CF, OF30, OF70, and OF100). Low-abundance taxa are grouped as “Unclassified.” Relative abundances were calculated based on OTU counts clustered at 97% sequence similarity.

**Figure S8. Overlap of fungi OTUs across fertilization treatments.** Venn diagram showing the overlap and uniqueness of fungi operational taxonomic units (OTUs; clustered at 97% sequence similarity) detected in rhizosphere soils under five fertilization regimes (CK, CF, OF30, OF70 and OF100). Numbers within each sector indicate the OTUs shared among specific combinations of treatments, while numbers in non-overlapping regions represent treatment-specific OTUs. Bar plots below the Venn diagram depict the total number of OTUs detected per treatment.


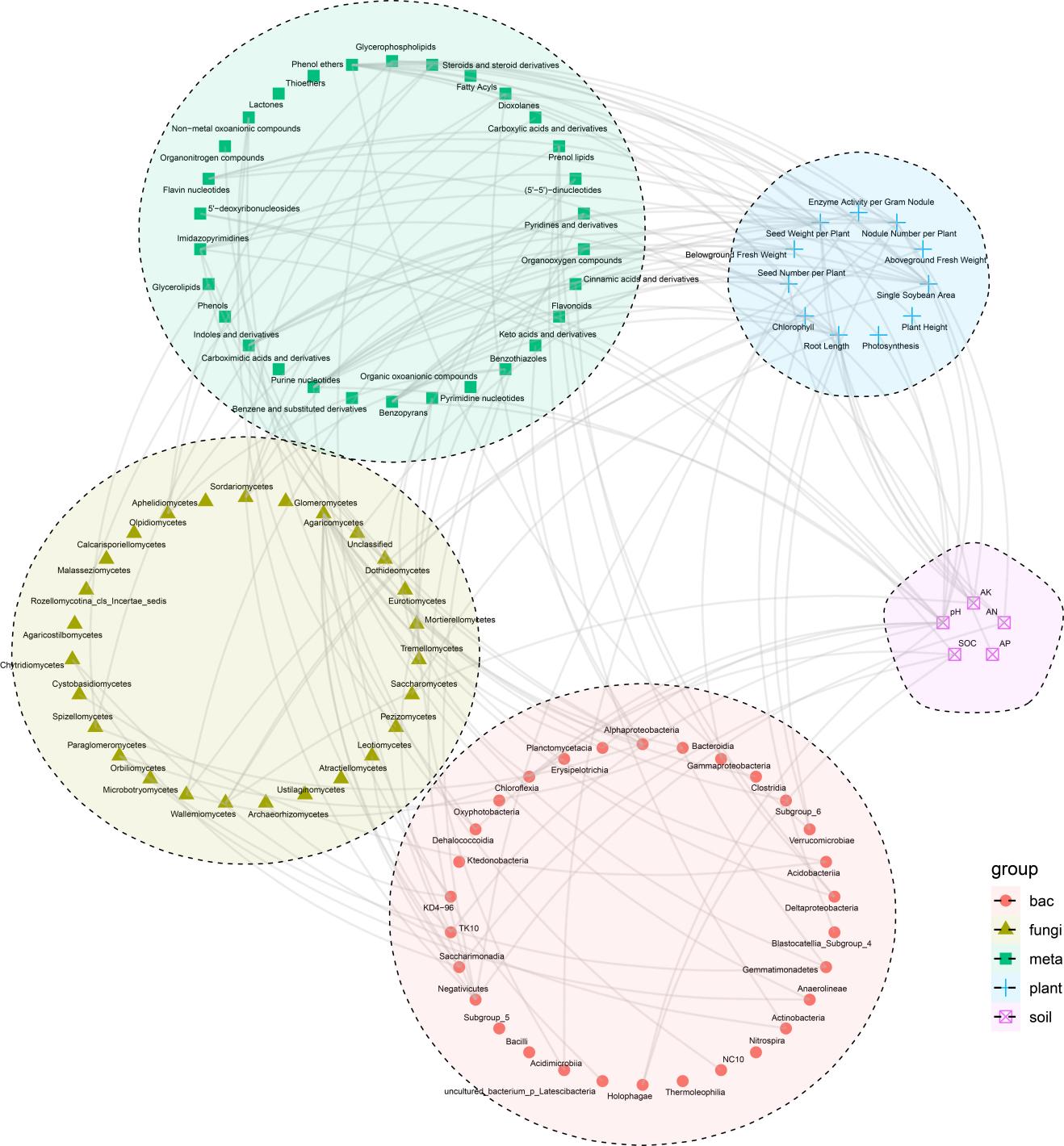


**Figure S9. Multi-omics network architecture linking rhizosphere microbiome, metabolite classes, soil properties, and soybean performance.** An integrated correlation network was constructed at the order level to resolve cross-kingdom associations among bacterial (red circles), fungal (yellow triangles), and metabolite classes (green squares), together with plant traits (blue crosses) and soil physicochemical variables (purple squares). Edges denote significant Spearman correlations (*P* < 0.05, |*r*| > 0.6). Dashed contours highlight major modules representing metabolite, fungal, bacterial, plant, and soil clusters.
